# Complex inter-relationship of body mass index, gender and serum creatinine on survival: exploring the obesity paradox in melanoma patients treated with checkpoint inhibition

**DOI:** 10.1186/s40425-019-0512-5

**Published:** 2019-03-29

**Authors:** Girish S. Naik, Sushrut S. Waikar, Alistair E. W. Johnson, Elizabeth I. Buchbinder, Rizwan Haq, F. Stephen Hodi, Jonathan D. Schoenfeld, Patrick A. Ott

**Affiliations:** 1 Department of Medical Oncology, Dana-Farber Cancer Institute, 450 Brookline Avenue, Boston, MA 02215 USA; 2 Harvard Medical School, Boston, MA USA; 3 Division of Renal Medicine, Department of Medicine, Brigham and Women’s Hospital, Boston, MA USA; 4 Institute of Medical Engineering & Science, Massachusetts Institute of Technology, Cambridge, MA USA; 5 Department of Radiation Oncology, Dana-Farber/Brigham and Women’s Cancer Center, Boston, MA USA; 6 Melanoma Center & Center for Immuno-Oncology, Boston, MA USA

**Keywords:** Anti-PD-1, Melanoma, Body mass index, Creatinine, Obesity paradox

## Abstract

**Background:**

A male gender driven obesity paradox (improved survival for overweight/obese patients compared to normal weight) was recently shown in melanoma in the context of checkpoint inhibition (anti-PD-1/anti-CTLA4 monotherapy) in a pooled meta-analysis. We characterized the relationship of Body Mass Index (BMI) with survival and explored gender-based interactions with surrogates of body composition/malnutrition in the context of PD-1 blockade as monotherapy or in combination with ipilimumab in a real-world setting.

**Methods:**

Advanced melanoma patients who received at least one dose of pembrolizumab, nivolumab, or nivolumab plus ipilimumab (combination) from June 2014 to September 2016 were included in this retrospective cohort study (*N* = 139). Overall Survival (OS) and Progression Free Survival (PFS) were the main outcomes. Analysis was performed using Random Survival Forests (RSF)/ multivariable Cox Proportional-Hazards models.

**Results:**

Overweight/Class-I (25- < 35 kg/m^2^) obese patients had a significantly lower risk of mortality (adjusted-HR:0.26; 95%CI:0.1–0.71; *p*-value = *0.008*) and progressive disease (adjusted-HR:0.43; 95%CI:0.19–0.95; *p*-value:*0.038*) compared to normal-weight (18.5- < 25 kg/m^2^). Class II/III obesity (compared to normal-weight) had an adjusted HR of 0.42 (95%CI: 0.1–1.77; *p*-value: 0.238) for OS and 1 (95%CI:0.34–2.94; *p*-value:0.991) for PFS. Exploration of interactions for OS showed that the association was predominantly driven by males (adjusted-HR_males_:0.11; 95%CI:0.03–0.4; adjusted-HR_females_: 0.56; 95%CI:0.16–1.89_;_ *p*-value_interaction_:*0.044*); the association was not seen in patients with serum creatinine< 0.9 mg/dL (adjusted-HR:0.43; 95%CI:0.15–1.24; *p*-value_interaction_:*0.020*), who were predominantly females. These observations were made in both the anti-PD-1 monotherapy (*n* = 79) and combination therapy (anti-PD-1/CTLA-4, *n* = 60) cohorts.

**Conclusions:**

The findings support the existence of an “obesity paradox” restricted to overweight/Class-I obesity in the real-world setting; the association was driven predominantly by males who largely had higher serum creatinine levels, a surrogate for skeletal muscle mass in the setting of metastatic disease. These observations suggest that sarcopenia (low skeletal muscle mass) or direct measures of body mass composition may be more suitable predictors of survival in melanoma patients treated with PD-1 blockade (monotherapy/combination).

**Electronic supplementary material:**

The online version of this article (10.1186/s40425-019-0512-5) contains supplementary material, which is available to authorized users.

## Background

Anti-Programmed Death-1 (PD-1) based immunotherapies have led to substantially improved survival in patients with advanced melanoma [1–4]. Combination therapy with ipilimumab and nivolumab results in higher response rates albeit with significant toxicity [5]. Despite these improved outcomes, many patients have either primary resistance or develop acquired resistance [6]. While tumor PD-L1 expression, mutational/neoantigen load, and IFN-γ signatures have some utility as predictors of outcome with checkpoint inhibitors [7–9], clinical predictors also may have value for the identification of patient subgroups who have longer survival and/or higher response rates to these therapies.

Nearly 70% of the adult population in the United States are either overweight or obese according to the CDC/National Center for Health Statistics and has reached epidemic proportions [10]. While obesity has generally been shown to pose a risk for many chronic diseases [11–13] as well as some solid cancers such as colon cancer, esophageal carcinoma and renal cell carcinoma [14], it has been associated with improved survival in other cancers including renal cell carcinoma [15], colorectal cancer [16] and non-small cell lung cancer [17]. Observation of improved survival outcomes among overweight/obese patients compared to normal weight patients has been referred to as the “obesity paradox” [18]. This observation has recently been documented in metastatic melanoma patients in the context of immune checkpoint inhibition as monotherapy (PD-1/PD-L1 or CTLA-4) and targeted therapy in a pooled meta-analysis, but not of chemotherapy [19]. In addition, the association was noted predominantly in males and gender-based differences in hormonal effects were proposed as one of the possible explanations.

Several hypotheses have been posed to explain the obesity paradox observed in cancer. Reverse causality has been commonly proposed, a phenomenon where previously overweight/obese patients are classified as normal weight at study baseline due to rapid cancer related weight loss [18]. Methodological reasons cited for explaining the paradox include detection bias, selection bias, less aggressive disease among obese patients [15], confounding and inability of BMI to distinguish well between adiposity and lean body mass [18, 20]. Many of the studies exploring the obesity paradox have limitations as differences in body mass composition (or surrogate markers), malnutrition (surrogate markers of malnutrition inflammation cachexia syndrome), lifestyle habits or the influence of preceding disease related weight loss on survival were not adequately accounted for [18].

We collected extensive data on baseline characteristics and characterized the relationship of pre-treatment BMI on overall survival, progression free survival and clinical benefit outcomes in the context of anti-PD-1 therapy either as monotherapy (pembrolizumab or nivolumab) or combination (nivolumab combined with ipilimumab) for advanced melanoma in a real-world setting and explored gender-based interactions with surrogates of body composition/malnutrition to understand potential factors driving the obesity paradox.

## Methods

### Patient population and study design

Unresectable or metastatic melanoma patients who received at least one dose of anti-PD-1 monotherapy (pembrolizumab or nivolumab) or combination (nivolumab plus ipilimumab) treatment from June 2014 to September 2016 outside of a clinical trial were included in this retrospective cohort study. Patients with brain metastases prior to the first dose of anti-PD-1 monotherapy or combination treatment were excluded. Prior exposure to investigational or approved immune checkpoint inhibitors (ICI) was not allowed except for prior anti-CTLA-4 therapy.

### Ethics statement

Approval for the study was obtained from the Dana-Farber/Harvard Cancer Center Institutional Review Committee.

### Outcomes

The primary outcome was Overall Survival (OS). OS was calculated from the date of first dose of ICI until the date of death. The outcomes of patients who did not have an event at the last follow up were censored. Secondary outcomes included progression free survival (PFS) and durable clinical benefit (DCB) as defined previously [8].

### Covariates

We retrospectively collected data from electronic health records. Baseline covariates included disease related characteristics, disease severity measures, co-morbidities, co-medications, lifestyle habits, clinical chemistry and blood count profiles. A total of 42 covariates/features were included: BMI, age, gender, race, melanoma type, BRAF V600 mutation, NRAS mutation, stage, Karnofsky Performance Score (KPS), Lactate Dehydrogenase (LDH), ever smokers, current drinkers, prior treatments (anti-CTLA-4/Immunotherapy, chemotherapy, radiation, targeted therapy), Charlson’s Comorbidity Index [21, 22], diabetes, hypertension (including baseline systolic and diastolic blood pressure), hyperlipidemia, chronic kidney disease (CKD) [23], cardiovascular disease (CAD/CHF/MI/AF), autoimmune/immune mediated disorders, co-medications (Anti-platelet agents such as aspirin/clopidogrel, anti-hypertensive medications (any), ACE inhibitors (Angiotensin Converting Enzyme) or ARBs (Angiotensin Receptor Blocker), metformin, statins and oral steroids), albumin, ANC (absolute neutrophil count), ALC (absolute lymphocyte count), hemoglobin, serum creatinine, eGFR (estimated glomerular filtration rate based on Cockcroft-Gault formula [23, 24]), fasting glucose, alkaline phosphatase, ALT (Alanine transaminase), AST (Aspartate transaminase), type of anti-PD-1 based immunotherapy (monotherapy/combination).

### Statistical analyses

Random Survival Forests (RSF) for survival outcomes and Random Forests (RF) for the binary outcome of DCB (See Additional file 1: Text) were used for analysis given distinct advantages of these methods in incorporating non-linear relationships, handling multi-collinearity, inclusion of covariate interactions and ability to handle high dimensional data under a non-parametric framework [25–29]. The adaptive nature of the forests and powerful visualization techniques which uncover complex patterns and inter-relationships/higher order interactions [25] with ease were of interest to elucidate the potential factors that could explain the obesity paradox in this treatment setting. The minimal depth criterion was used to assess the importance of BMI among other features studied. The threshold for minimal depth filtering was minimal depth below the mean value (Refer Additional file 1: Text). A total of 42 features deemed clinically important by expert knowledge (listed above) were assessed in the RSF and RF models. The number of features included for each individual tree was set at 7 and 2000 trees were grown in the forest. No feature selection was performed.

Partial dependence (adjusted risk estimate) plots were used to assess the relationship of BMI as a continuous variable on survival and clinical benefit outcomes. Subsequently, interactions were assessed by co-plots plotted as heat maps for the adjusted risk estimate either risk/mortality for survival outcomes and probability of achieving DCB for binary outcome (adjusted for remaining features in the model; Refer Additional file 1: Text for details on methodology). Two-way, three-way and four-way interactions were explored for features of importance and clinically relevant features as deemed by expert clinical knowledge (including surrogates of body composition/malnutrition and stratified by monotherapy/combination), by examining partial dependence plots (for individual features) and baseline characteristics to find potential explanations or mechanisms for the observed relationship of BMI with outcomes. Missing covariate data was handled by forest imputation for analyses in RSF and RF. Discrimination of the model was evaluated using the average concordance index (C-index) within a 10-fold cross-validation scheme. RSF has previously been used to help explain paradoxical findings of association of high BMI and improved survival in cardiovascular disease [25]. The packages used were “RandomForestsSRC”, “ggRandomForestsSRC”, “mlr”, “survminer”, “nephro” and “ggplot2” in R version 3.3.3 [30].

Results were confirmed by univariate and multi-variable Cox-PH models for survival outcomes and logistic regression for durable clinical benefit outcome (baseline characteristics with imbalance across BMI groups or covariates deemed clinically important in the context of studying BMI on survival outcomes were adjusted for; see Table 2 footnote). No imputation was performed for missing covariate data for multi-variable Cox-PH/logistic regression (complete case analysis).

### Sensitivity analyses

BMI measured within the past three to six months (the earliest measure available within this window period) was analyzed in place of BMI measured at baseline (pretreatment BMI just prior to receiving anti-PD-1 based immunotherapy) by RSF/RF to assess if the results and/or conclusions obtained were similar to primary analysis with pre-treatment BMI, to account for patients crossing over to the lower weight category due to disease-related or other causes of weight loss. The CKD-EPI equation [31] based eGFR as a continuous covariate was included instead of Cockcroft-Gault formula to assess if the findings were similar. Additionally, separate analyses for monotherapy/combination were performed.

## Results

A total of 139 patients (79 patients treated with anti-PD-1 monotherapy and 60 patients treated with ipilimumab plus nivolumab) were included in the analysis after reviewing 263 patients who had received PD-1 based therapies (excluded patients had received treatment as part of interventional trial protocols that were not eligible for this retrospective cohort (*n* = 63) or patients with brain metastases at baseline (*n* = 61)). Patients were mostly Caucasians and had cutaneous melanoma; 72% of patients were overweight or obese (BMI ≥ 25 kg/m^2^), a proportion that is comparable to the US population distribution of BMI [10]. The overweight/Class I obese group included a higher proportion of males, diabetes, hypertension, hyperlipidemia as well as use of anti-hypertensives, ACE/ARB inhibitors, and statins compared to patients with BMI < 25 kg/m^2^ (Table 1). Moreover, the overweight/Class I obese group had higher mean serum creatinine concentrations at baseline compared to patients with BMI < 25 kg/m^2^ and Class II/III obese patients (Table 1).

**Table 1:** Baseline characteristics by BMI for patients treated with PD-1 blockade

*N* = 139	Body Mass Index (kg/m^2^)		
	BMI < 25 kg/m^2^ *N* = 38^a^(%)	Overweight and Class I Obesity (BMI 25- < 35 kg/m^2^) *N* = 86 (%)	Class II/III Obesity (BMI ≥ 35 kg/m^2^) *N* = 15 (%)
Demographics			
Age	64.6 (15.2)	61.2 (13.8)	57.3 (13.7)
Male gender	19 (50)	57 (66.3)	3 (20)
White race	37 (97.4)	85 (98.8)	14 (93.3)
Cutaneous Melanoma	31 (81.6)	78 (90.7)	12 (80)
BRAF V600 mutation	11 (28.9)	22 (25.6)	2 (13.3)
NRAS mutation	4 (10.5)	6 (7)	4 (26.7)
Disease severity			
Stage at baseline	IV M1c: 24 (63.2)	IV M1c: 49 (57)	IV M1c: 7 (46.7)
Karnofsky Performance Score (KPS)	< 70: 2 (5.3) > = 70: 36 (94.7)	< 70: 2 (2.4) > = 70: 83 (97.6) *N* = 85/86	< 70: 0 (0) > = 70: 15 (100)
LDH in U/L (Median and IQR; *N* = 131)	211.5 (158–336) *N* = 34/38	176.5 (137–230) (*N* = 82/86)	187 (158–264)
Lifestyle habits			
Ever smokers	Former: 16 (42.1) Current: 3 (7.9)	Former: 34 (39.5) Current: 8 (9.3)	Former: 7 (46.7) Current: 0 (0)
Current drinkers	24 (63.2)	54 (62.8)	6 (40)
Prior treatments			
Immunotherapy	9 (23.7)	33 (38.4)	8 (53.3)
Chemotherapy	1 (2.6)	12 (14)	2 (13.3)
Radiation	10 (26.3)	22 (25.6)	6 (40)
Targeted therapy	5 (13.2)	5 (5.8)	0 (0)
Co-morbidities			
Charlson’s Comorbidity Index (Mean and SD)	8.6 (1.7)	8.4 (2.1)	8.1 (1.7)
Diabetes	3 (7.9)	11 (12.8)	4 (26.7)
Hypertension	14 (36.8)	51 (59.3)	9 (60)
Hyperlipidemia	11 (28.9)	27 (31.4)	8 (53.3)
Chronic Kidney Disease (CKD)	3 (7.9)	7 (8.1)	1 (6.7)
Cardiovascular disease (CAD/CHF/MI/AF)^c^	6 (15.8)	11 (12.8)	4 (26.7)
Autoimmune/Immune mediated disorders	7 (18.4)	9 (10.5)	1 (6.7)
Co-medications for co-morbidities			
Anti-platelet agents (Aspirin/Clopidogrel)	10 (26.3)	22 (25.6)	4 (26.7)
Anti-hypertensive medications (any)	10 (26.3)	49 (57)	9 (60)
ACE or ARB inhibitors	7 (18.4)	28 (32.6)	4 (2.7)
Metformin	3 (7.9)	6 (7)	3 (20)
Statins	11 (2.9)	20 (23.3)	4 (26.7)
Oral Steroids	1 (2.6)	10 (11.6)	1 (6.7)
Clinical chemistry and Vitals			
Albumin in g/dL (Mean and SD)	3.8 (0.59)	4.1 (0.41)	4 (0.45)
ANC K/uL (Mean and SD)	5.5 (3.3)	4.8 (1.6)	5.5 (2.7)
ALC K/uL (Mean and SD)	1.3 (0.6)	1.6 (1.4)	1.8 (1.1)
Hemoglobin g/dL (Mean and SD)	12.4 (2)	13.1 (1.6)	12.2 (1.5)
Serum Creatinine mg/dL (Mean and SD)	0.81 (0.21)	0.95 (0.38)	0.88 (0.36)
eGFR^b^ (ml/min/1.73m^2^)	> = 60: 36; < 60: 2	> = 60: 77; < 60: 9	> = 60: 13; < 60: 2
eGFR by CKD-EPI equation in ml/min/1.73m^2^ (Median and IQR)	88.51 (71.09–98.91)	85.87 (74.83–94.73)	91.03 (70.99–100.6)
Fasting Glucose in mg/dL (Median and IQR)	106 (100–122)	101 (93–119)	102 (98–114)
BMI at baseline (kg/m^2^)	22.6 (2.3)	29.1 (2.7)	40.1 (4.4)
Alkaline Phosphatase in U/L (Median and IQR)	74 (63–100)	74 (62–90)	69 (60–129)
ALT in U/L (Median and IQR)	15 (11–27)	17 (12–21)	18 (14–27)
AST in U/L (Median and IQR)	19 (15–29)	18 (13–23)	21 (13–27)
Systolic blood pressure in mm Hg (Mean/SD)	124.7 (19.9)	133.8 (18.2)	135.3 (14.4)
Diastolic blood pressure in mm Hg (Mean/SD)	74.4 (11.9)	78.3 (13.2)	75 (10.6)
Disease related weight loss			
BMI measured up to 6 months before baseline (Mean and SD)	23 (2.6) (*N* = 35/38)	29.3 (2.7) (*N* = 81/86)	40.4 (4.8)
Treatment			
Anti-PD-1 immunotherapy type	Mono: 19 (50) Combination:19 (50)	Mono: 49 (57) Combination: 37 (43)	Mono: 11 (73.3) Combination: 4 (26.7)

^a^Includes three patients with BMI < 18.5 for descriptive purposes. Analyses by Cox-PH/logistic regression was performed by excluding underweight patients (*n* = 3) but were included for RSF analysis where BMI was included as a continuous variable

^b^*eGFR* Estimated Glomerular Filtration Rate (Cockcroft-Gault)

^c^*CAD* Coronary Artery Disease, *CHF* Congestive Heart Failure, *MI* Myocardial Infarction and *AF* Atrial Fibrillation

*IQR* Inter Quartile Range

**Fig. 1:**
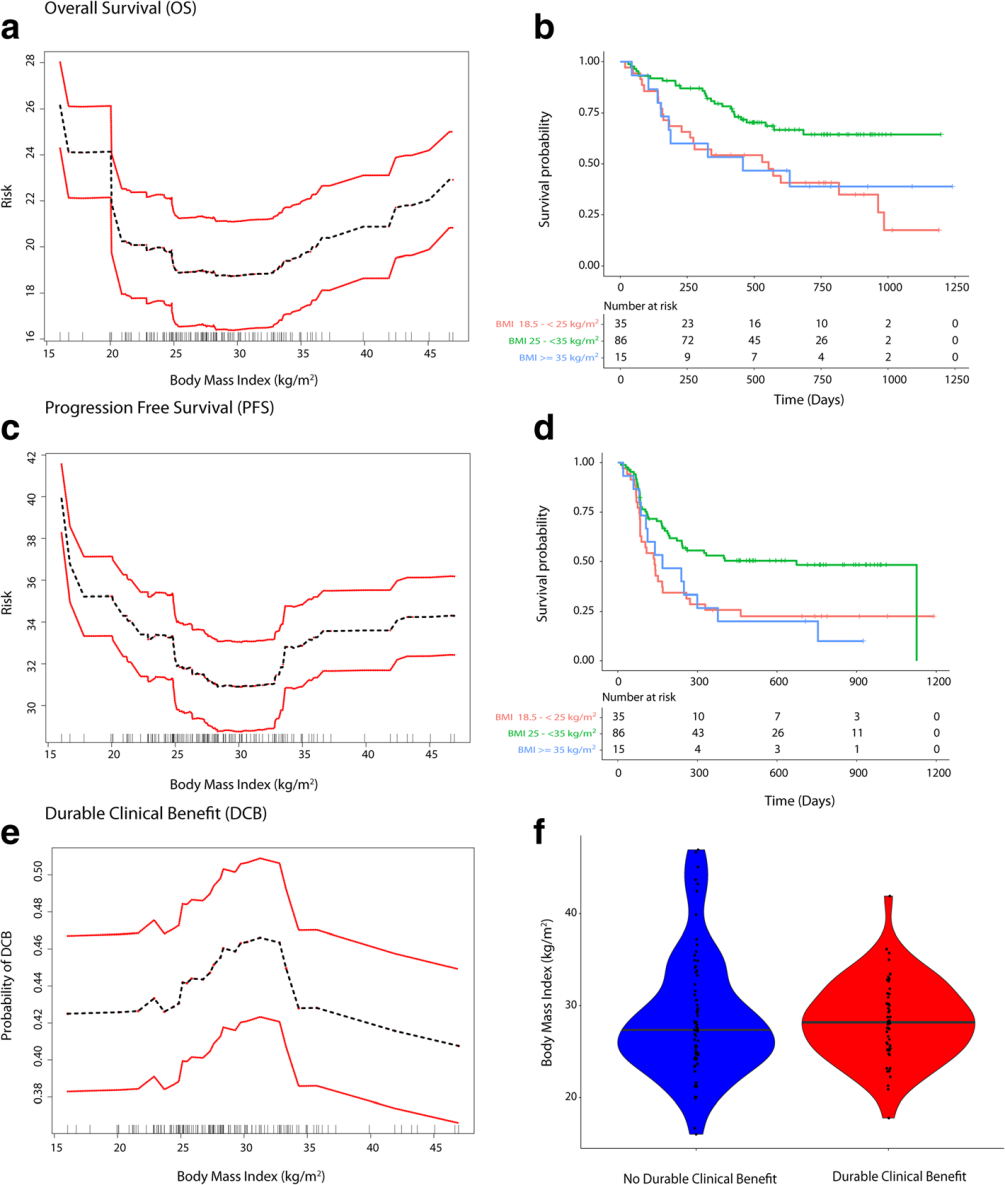
Relationship of BMI with survival and clinical benefit (**a**-**f**). Panel **a** shows the relationship between BMI and risk (mortality), with the risk being the lowest for overweight/Class I obese patients and the highest for underweight/normal weight patientsand panel **b** shows the KM plots for the identified BMI risk groups. Panel **c** and **d** shows similar findings for PFS. Panel **e** shows the relationship for durable clinical benefit outcome. Panel **f** shows the distribution of BMI in patients with and without durable clinical benefit. Note: Figures **b** and **d** excluded 3 underweight patients

After a median follow up of 760 days (IQR: 522–910), 25/38 patients with BMI < 25 had died (1-year event-rate: 51.6% (95%CI: 34.9–76.4%)), 27/86 patients who were overweight/Class I obese had died (1-year event-rate: 20.9% (95%CI: 14.3–30.5%)) and 9/15 patients who were Class II/Class III obese had died (1-year event-rate: 43.3% (95%CI: 22.5–83.2%)). The median OS was not reached (NR) for overweight/Class I obese patients. Median OS was 530 days (IQR: 157 days – 985 days) for patients with BMI < 25 kg/m^2^ and was 458 days (IQR: 152 days – NR) for Class II/III obese patients. The median PFS was 673 days (IQR: 109–1126) for overweight/Class I obese patients, was 135 days (IQR: 75 days – 463 days) for patients with BMI < 25 kg/m^2^ and was 168 days (IQR: 87 days – 377 days) for Class II/III obese patients. The median OS was not reached for males (*n* = 79) and was 554 days (IQR: 273–985 days) for females (*n* = 60) and the one-year event rate for males was 24.6% (95%CI: 17.1–35.4%) and 39.8% (95%CI: 28.2–56.3%) for females. The KM survival curves (OS and PFS) are shown in Fig. 1b, d. The KM survival graphs stratified by treatment and gender are shown in Additional file 1: Figure S1.

### RSF/RF findings

Minimal depth statistic in RSF deemed BMI as predictive based on minimal depth (lower than the mean; KPS was the strongest predictor; Refer to Additional file 1: Text). The mean C-index was 0.80 for OS. Partial dependence plots in RSF analysis showed a “U” shaped relationship of pretreatment BMI and risk of mortality as well as progressive disease and an “inverted U” shaped relationship for probability of achieving DCB (Fig. 1a, c and e). Partial dependence-derived cutoffs/inflection points corresponded to WHO-based BMI categories wherein, overweight or Class I (25- < 35 kg/m^2^) obese patients had a lower predicted risk of mortality and disease progression and higher probability of achieving DCB compared to normal BMI (18.5- < 25 kg/m2) and Class II/III obese patients (≥35 kg/m^2^). Underweight/normal weight patients had the highest risk of mortality and progressive disease (Fig. 1a, c and e).

### Exploration of interactions

**Fig. 2:**
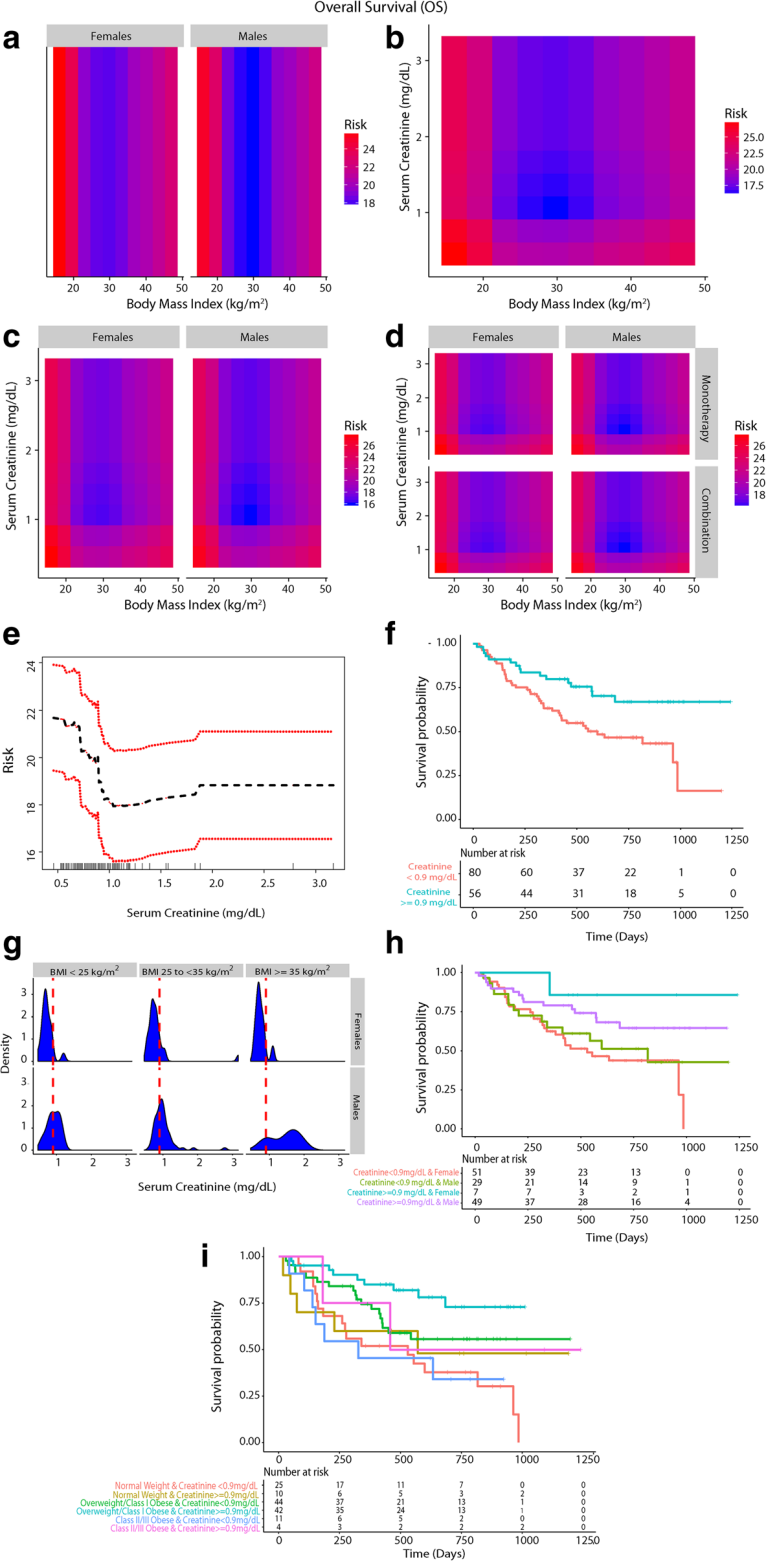
Inter-relationship of BMI, gender, serum creatinine and OS (**a**-**i**). Panel A shows the predominant male gender driven association of overweight/Class I obesity with lower risk of mortality (dark blue) compared to normal weight/underweight patients and Class II/III obese patients who had higher risk of mortality (red). Panel **b** shows that patients who had serum creatinine < 0.9 mg/dL had high risk of mortality and the obesity paradox pattern (blue) was largely attenuated. Panel **c** shows that the obesity paradox finding was attenuated for both genders if serum creatinine concentrations were < 0.9 mg/dL. Panel **d** shows that findings from Panel **c** were noted for both treatments (monotherapy/combination). Panel **e** shows an “L” shaped relationship of serum creatinine with OS. Panel **f** shows KM survival curves for the two creatinine risk groups per RSF thresholds (excluding 3 underweight patients). Panel **g** shows gender-based differences in distribution of serum creatinine within BMI groups where most females had serum creatinine < 0.9 mg/dL (risk threshold identified by RSF and is indicated as a red dashed line). Panel **h** shows that patients with serum creatinine ≥0.9 mg/dL who had longer survival were predominantly males than patients with serum creatinine < 0.9 mg/dL who were predominantly females. Panel **i** shows that overweight/Class I obese patients with serum creatinine > = 0.9 mg/dL had the longest OS

**Fig. 3:**
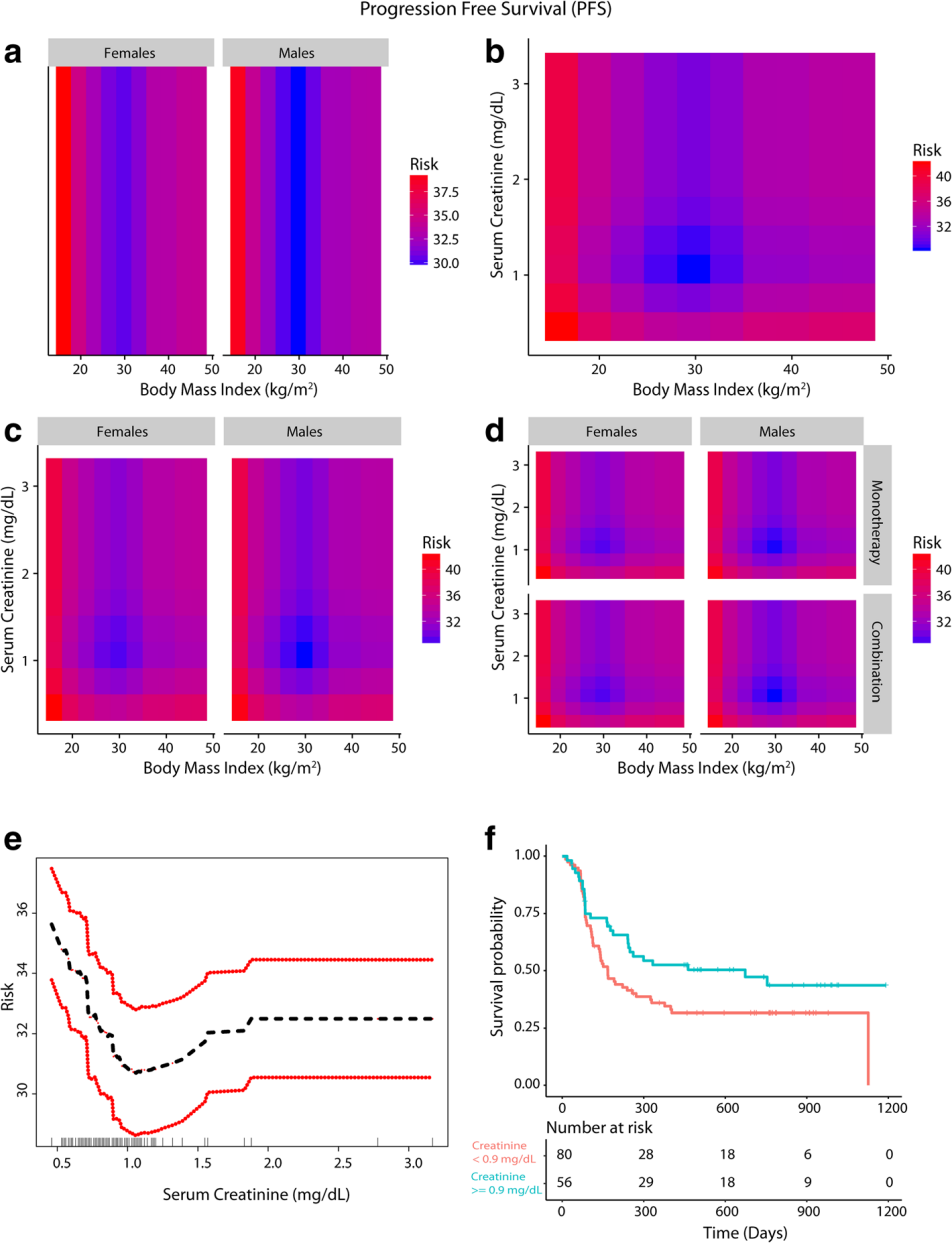
Inter-relationship of BMI, gender, serum creatinine and PFS (**a**-**f**). Panel **a** shows the predominant male gender driven association of overweight/Class-I obesity with lower risk of progressive disease (dark blue) compared to normal weight/underweight patients and Class-III obese patients who had higher risk of disease progression (red). Panel **b** shows that patients who had serum creatinine < 0.9 mg/dL had high risk of progressive disease and the obesity paradox pattern (blue) was largely attenuated. Panel **c** shows that for both genders the paradox was attenuated if serum creatinine was < 0.9 mg/dL. Panel **d** shows that the findings from Panel **c** were noted both for anti-PD-1 based monotherapy and combination therapy. Panel **e** shows the relationship of serum creatinine with PFS and Panel **f** shows improved PFS for patients with serum creatinine > = 0.9 mg/dL compared to patients with levels < 0.9 mg/dL

**Fig. 4:**
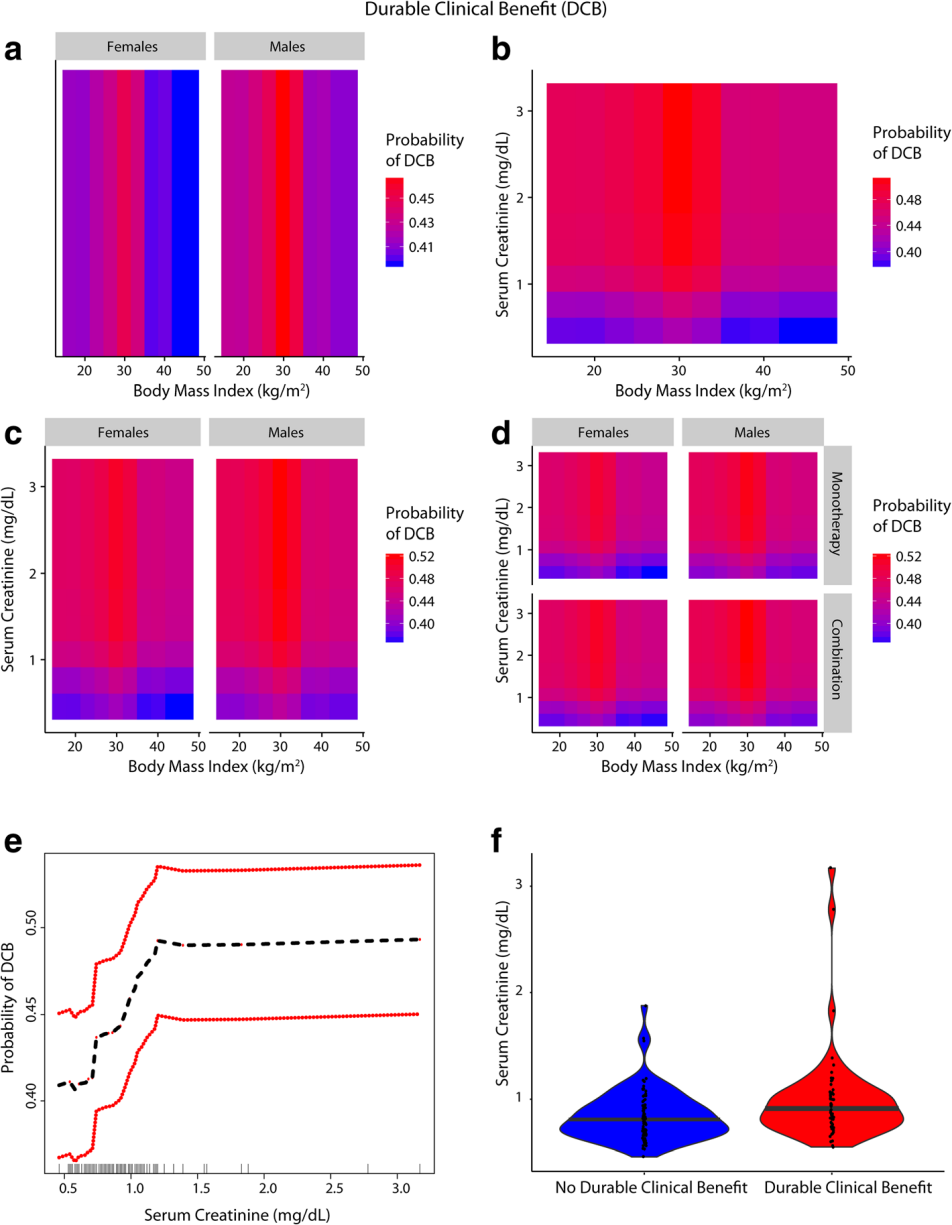
Inter-relationship of BMI, gender, serum creatinine and Durable Clinical Benefit (DCB) (**a**-**f**): Panel **a** shows the predominant male gender driven association of overweight/Class-I obesity with a higher probability of achieving DCB (red) compared to normal weight/underweight patients and Class-II/III obese patients who had lower probability of DCB (blue). Panel **b** shows that patients who had serum creatinine < 0.9 mg/dL had lower probability of DCB and the obesity paradox pattern (red) was largely attenuated. Panel **c** shows that the paradox was attenuated for both genders for lower serum creatinine levels (< 0.9 mg/dL). Panel **d** shows that findings from Panel **c** were noted for both anti-PD-1 based monotherapy and combination therapy. Panel **e** shows the relationship of serum creatinine with probability of achieving DCB. Panel **f** shows the distribution of serum creatinine among patients with and without DCB

A gender driven difference in survival and clinical benefit outcomes was apparent in co-plots, where overweight/Class I obese males had a lower predicted risk of mortality/progressive disease than overweight/Class I obese females (Figs. 2a, 3a and 4a). Further, there was an interaction between BMI and serum creatinine such that the obesity paradox was attenuated for the subgroups of patients with serum creatinine levels < 0.9 mg/dL (Figs. 2b, 3b and 4b; Additional file 1: Figure S2). Relationship of BMI, serum creatinine and gender on survival and durable clinical benefit outcomes revealed that the obesity paradox was attenuated for both genders in patients with serum creatinine < 0.9 mg/dL (Figs. 2c, 3c and 4c). These findings were noted for both mono- and combination therapy (Figs. 2d, 3d and 4d). Serum creatinine was deemed predictive based on minimal depth criteria while the minimal depth for gender was above the threshold for minimal depth filtering criterion). Examination of the partial dependence of serum creatinine on OS revealed an “L” shaped relationship with survival outcome where patients with creatinine levels < 0.9 mg/dL had a high risk of mortality and levels lower than 0.7 mg/dL had the highest risk of mortality (Fig. 2e and f). Gender-based density distribution of serum creatinine within the three BMI risk groups showed that most females had serum creatinine < 0.9 mg/dL (Fig. 2g). Baseline characteristics (Additional file 1: Table S1) grouped by serum creatinine (< and ≥ 0.9 mg/dL) showed that only 13.3% of patients with serum creatinine ≥0.9 mg/dL were females whereas 87.7% were males and prolonged OS was noted among patients with serum creatinine ≥0.9 mg/dL who were mostly males (Fig. 2h). Overweight/Class I obese patients with serum creatinine ≥0.9 mg/dL had the longest survival (Fig. 2i).

Exploration of other gender-based interactions of BMI and serum creatinine with co-morbidities, lifestyle habits, co-medications for co-morbidities, BRAF V600, NRAS mutations, prior therapies for melanoma (targeted therapy, immunotherapy, chemotherapy and radiation) and interaction of BMI with clinical chemistry parameters, disease severity and other demographics did not provide plausible strong alternative explanations for gender-based difference (Additional file 1: Text, Figures S3-S5 and Table S1).

### Cox-PH/logistic regression and sensitivity analyses

**Fig. 5:**
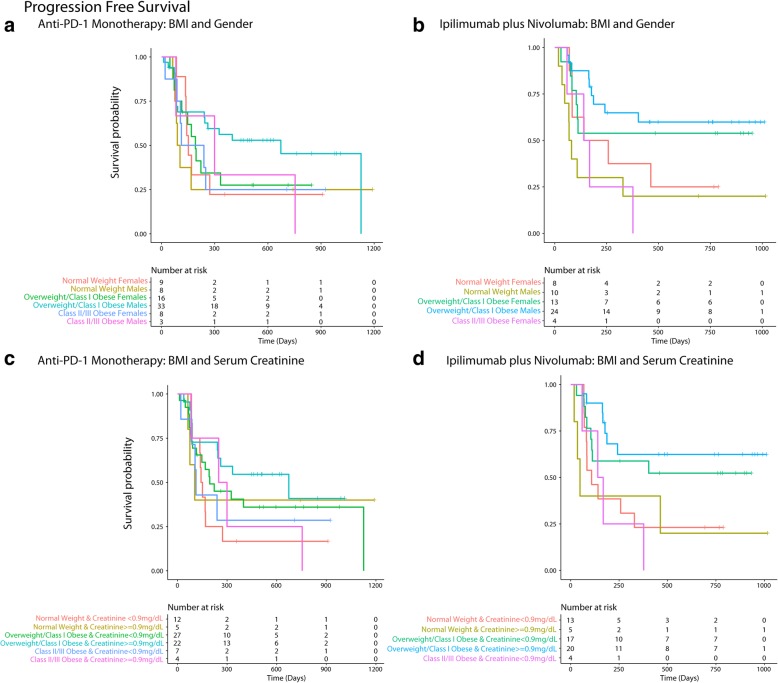
Panel **a** and **b** shows that overweight/ Class 1 obese males had the longest progression free survival (PFS) among patients treated with monotherapy (**a**) and combination (**b**). Panel **c** and **d** shows that overweight/Class 1 obese patients with serum creatinine > = 0.9 mg/dL had the longest PFS among patients treated with monotherapy (**c**) and combination (**d**)

**Fig. 6:**
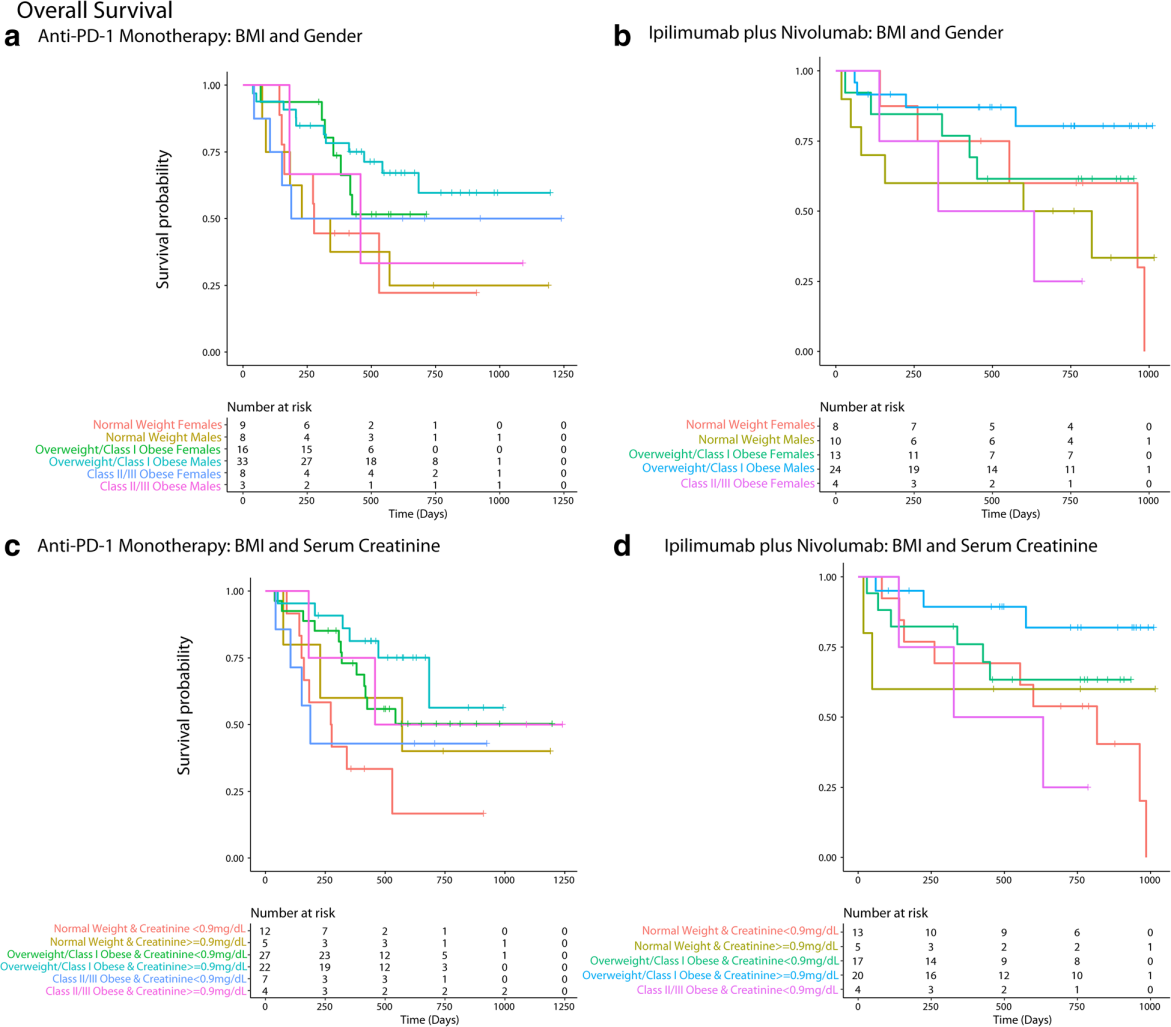
Panel **a** and **b** shows that overweight/ Class 1 obese males had the longest overall survival (OS) among patients treated with monotherapy (**a**) and combination (**b**). Panel **c** and **d** shows that overweight/Class 1 obese patients with serum creatinine > = 0.9 mg/dL had the longest OS among patients treated with monotherapy (**c**) and combination (**d**)

The findings from Cox-PH and logistic regression (univariate and multi-variable) largely supported the results from RSF/RF (Table 2). BMI measured within 3 to 6 months showed a similar relationship for survival outcomes as for pre-treatment BMI measured just prior to first dose of anti-PD-1 immunotherapy (Additional file 1: Figure S6). Primary findings were similar when CKD-EPI based eGFR was used in RSF analysis in place of Cockcroft-Gault based eGFR (Additional file 1: Figure S7). Effect estimates for monotherapy and combination therapies are given separately in Table 3 supporting the findings noted in Figs. 5, 6 and Table 2.

**Table 2:** Hazard ratios for the association of BMI with OS and PFS (along with interactions) and odds ratios for the association of BMI with durable clinical benefit among patients treated with anti-PD-1 based checkpoint inhibition

Outcome (*N* = 136)^b^	Effect Estimate			
Overall Survival (OS)	Unadjusted Hazard Ratio (95% CI)	*p-value*	Adjusted Hazard Ratio^a^ (95% CI)	*p-value*
Overweight/Class-I Obese vs. Normal Weight (reference)	0.41 (0.24–0.72)	*0.002*	0.26 (0.1–0.71)	*0.008*
Class-II/III Obese vs. Normal Weight	0.88 (0.41–1.91)	0.756	0.42 (0.1–1.77)	0.238
Interaction Model^c^ 1 (OS)				
Interaction of BMI (Overweight/Class-I vs. normal weight) with gender (males vs. females)	–	–	0.19 (0.04–0.95)	*0.044*
Interaction Model 1 (OS)				
Overweight/Class-I Obese (vs. normal weight) HR in females	–	–	0.56 (0.16–1.89)	0.346
Interaction Model 1 (OS)				
Overweight/Class-I Obese (vs. normal weight) HR in males	–	–	0.11 (0.03–0.4)	*0.001*
Interaction Model 2 (OS)				
Interaction of BMI (Overweight/Class-I vs. normal weight) with serum creatinine (> = 0.9 mg/dL vs. < 0.9 mg/dL)^c^	–	–	0.11 (0.02–0.7)	*0.020*
Interaction Model 2 (OS)				
Overweight/Class-I Obese (vs. normal weight) HR in patients with serum creatinine < 0.9 mg/dL	–	–	0.43 (0.15–1.24)	0.119
Interaction Model 2 (OS)				
Overweight/Class-I Obese (vs. normal weight) HR in patients with serum creatinine > = 0.9 mg/dL	–	–	0.045 (0.08–0.262)	*0.001*
Progression Free Survival (PFS)				
Overweight/Class-I Obese vs. Normal Weight (reference)	0.5 (0.31–0.81)	*0.005*	0.43 (0.19–0.95)	*0.038*
Class-II/III Obesity vs. Normal Weight	1.03 (0.53–2)	0.932	1 (0.34–2.94)	0.991
Interaction Model 1 (PFS)				
Interaction of BMI (overweight/Class-I vs. normal weight) with gender (males vs. females)	–	–	0.29 (0.07–1.18)	0.084
Interaction Model 1 (PFS)				
Overweight/Class-I Obese (vs. normal weight) HR in females	–	–	0.80 (0.27–2.41)	0.695
Interaction Model 1 (PFS)				
Overweight/Class-I Obese (vs. normal weight) HR in males	–	–	0.23 (0.08–0.66)	*0.006*
Interaction Model 2 (PFS)				
Interaction of BMI (overweight/Class-I vs. normal weight) with serum creatinine (> = 0.9 mg/dL vs. < 0.9 mg/dL)	–	–	0.17 (0.04–0.77)	*0.021*
Interaction Model 2 (PFS)				
Overweight/Class-I Obese (vs. normal weight) HR in patients with serum creatinine < 0.9 mg/dL	–	–	0.71 (0.29–1.77)	0.464
Interaction Model 2 (PFS)				
Overweight/Class-I Obese (vs. normal weight) HR in patients with serum creatinine > = 0.9 mg/dL	–	–	0.12 (0.03–0.45)	*0.002*
Durable Clinical Benefit (*N* = 132)^b^	Unadjusted Odds Ratio (95% CI)	*p-value*	Adjusted Odds Ratio^d^ (95% CI)	*p-value*
Overweight/Class-I Obese vs. Normal Weight (reference)	2.76 (1.18–6.46)	*0.020*	11.4 (1.65–78.6)	*0.013*
Class-II/III Obesity vs. Normal Weight	0.91 (0.23–3.54)	0.891	3.3 (0.22–50.5)	0.391
Interaction Model 1 (DCB)				
Interaction of BMI (overweight/Class-I vs. normal weight) with gender (males vs. females)	–	–	10.39 (0.17–634.68)	0.265
Interaction Model 1 (DCB)				
Overweight/Class-I Obese (vs. normal weight) in females	–	–	2.91 (0.16–54.05)	0.473
Interaction Model 1 (DCB)				
Overweight/Class-I Obese (vs. normal weight) in males	–	–	30.27 (2.01–455.72)	*0.014*
Interaction Model 2 (DCB)				
Interaction of BMI (overweight/Class-I vs. normal weight) with creatinine (> = 0.9 mg/dL vs. < 0.9 mg/dL)	–	–	2.82 (0.03–229.67)	0.644
Interaction Model 2 (DCB)				
Overweight/Class-I Obese (vs. normal weight) HR in patients with serum creatinine < 0.9 mg/dL	–	–	8.03 (0.85–76.27)	0.070
Interaction Model 2 (DCB)				
Main effect (Serum Creatinine > = 0.9 mg/dL): Overweight/Class-I Obese (vs. normal weight) HR in patients with serum creatinine > = 0.9 mg/dL	–	–	22.66 (0.51–1013.5)	0.108

^a^ Adjusted for the following covariates: age (<=45, > 45–75 and > 75 years), gender, serum creatinine (< 0.9 and > = 0.9 mg/dL), treatment (monotherapy/combination), current drinker (vs. non-current/never-drinker), smoking history (ever vs. never), KPS (<=70 and > 70), LDH (<=231 vs. > 231 U/L), stage at baseline, Charlson’s score (< 10 vs. > = 10), hemoglobin (< 11.5 vs. > = 11.5 g/dL), ANC (<=8 and > 8 K/uL), ALC (< 3 and > =3 K/uL), albumin (< 3.5 g/dL), autoimmune disease, diabetes, CV disease, CKD, BRAF mutation, NRAS mutation, hypertension, hyperlipidemia, comedications (anti-platelet agents, statins, metformin, ACE/ARB inhibitors), prior treatments (immunotherapy/CTLA-4, radiation, chemotherapy and targeted therapy), fasting glucose (<=110 vs. > 110 mg/dL), type of melanoma,; *N* = 127 after excluding three patients who were underweight, 8 patients with missing LDH values and 1 patient whose KPS could not assessed (missing)

^b^ Three underweight patients were excluded

^c^ Interactions were studied in separate models along with main effects and adjusted for the same covariates as the main models

^d^ Adjusted for the same covariates listed for OS and PFS (except KPS which was included as a continuous variable); *N* = 123 (excluding 3 underweight patients; DCB was not assessable/available for 4 patients; patients with missing data for LDH, KPS were not included for the adjusted analysis)

**Table 3:** Effect estimates for the association of BMI and gender, BMI and serum creatinine on PFS, OS and DCB outcomes by anti-PD1 monotherapy and combination (ipilimumab plus nivolumab)

Outcome: PFS	
Type of checkpoint inhibition	Effect Estimate^a^: HR (95% CI)
Anti-PD1 Monotherapy (*N* = 77)	
BMI and Gender (Overweight/Class 1 Obese Males vs. Normal Weight Females)	0.567 (0.233–1.378)
BMI and Gender (Overweight/Class 1 Obese Males vs. Normal Weight Males)	0.509 (0.196–1.323)
BMI and Serum Creatinine (Overweight/Class 1 Obese & Serum Creatinine > = 0.9 mg/dL vs. Normal Weight & Serum Creatinine < 0.9 mg/dL)	0.439 (0.184–1.047)
Ipilimumab plus Nivolumab (*N* = 59)	
BMI and Gender (Overweight/Class 1 Obese Males vs. Normal Weight Females)	0.424 (0.151–1.192)
BMI and Gender (Overweight/Class 1 Obese Males vs. Normal Weight Males)	0.243 (0.093–0.632)
BMI and Serum Creatinine (Overweight/Class 1 Obese & Serum Creatinine > = 0.9 mg/dL vs. Normal Weight & Serum Creatinine < 0.9 mg/dL)	0.316 (0.120–0.835)
Outcome: OS	
Type of checkpoint inhibition	Effect Estimate: HR (95% CI)
Anti-PD1 Monotherapy (*N* = 77)	
BMI and Gender (Overweight/Class 1 Obese Males vs. Normal Weight Females)	0.331 (0.121–0.903)
BMI and Serum Creatinine (Overweight/Class 1 Obese & Serum Creatinine > = 0.9 mg/dL vs. Normal Weight & Serum Creatinine < 0.9 mg/dL)	0.208 (0.073–0.591)
Ipilimumab plus Nivolumab (*N* = 59)	
BMI and Gender (Overweight/Class 1 Obese & Males vs. Normal Weight Females)	0.294 (0.078–1.098)
BMI and Serum Creatinine (Overweight/Class 1 Obese & Serum Creatinine > = 0.9 mg/dL vs. Normal Weight & Serum Creatinine < 0.9 mg/dL)	0.208 (0.056–0.768)
Outcome: DCB	
Type of checkpoint inhibition	Effect Estimate: OR (95%CI)
Anti-PD1 Monotherapy (*N* = 75)	
BMI and Gender (Overweight/Class 1 Obese & Males vs. Normal Weight Females)	5.115 (0.914–28.640)
BMI and Serum Creatinine (Overweight/Class 1 Obese & Serum Creatinine > = 0.9 mg/dL vs. Normal Weight & Serum Creatinine < 0.9 mg/dL)	7.22 (1.268–41.143)
Ipilimumab plus Nivolumab (*N* = 57)	
BMI and Gender (Overweight/Class 1 Obese & Males vs. Normal Weight Females)	2.407 (0.456–12.720)
BMI and Serum Creatinine (Overweight/Class 1 Obese & Serum Creatinine > = 0.9 mg/dL vs. Normal Weight & Serum Creatinine < 0.9 mg/dL)	2.813 (0.627–12.611)

^a^All effect estimates are un-adjusted

## Discussion

### Nonlinear relationship of BMI with survival and inter-relationship of BMI and serum creatinine levels

Patients with normal or below normal weight were at higher risk of poor survival outcomes and less likely to achieve durable clinical benefit compared to overweight/Class I obese patients. The protective association noted with overweight/Class I obese patients compared to normal weight group was not observed for Class II/III obesity but the sample size in this group was limited (Fig. 1 and Table 2). These findings confirm the presence of an “obesity paradox” recently reported in advanced melanoma patients treated with immune checkpoint inhibition (anti-PD-1/anti-CTLA-4 monotherapy) or targeted therapy in a pooled meta-analysis [19], albeit for overweight/Class I obesity in this study. The survival curves closely overlapped for overweight and obese patients for the PD-1 monotherapy cohort in the study by McQuade et al. [19], but the study did not further classify patients based on the three classes of obesity. The findings suggest that relationship of BMI with survival is likely dependent on underlying body mass composition. The observed relationship of BMI with survival in our study likely indicates two themes. First, low lean body mass is a risk factor for poor outcome and second, high adiposity is also associated with adverse outcome; both observations have been documented previously in other cancers including colorectal cancer [16, 32, 33]. However, there is a concern that BMI does not accurately indicate either lean mass or adiposity [34]. Recent studies have shown the importance of assessing skeletal muscle mass/body mass composition to predict survival in cancers as even obese patients can have underlying sarcopenia (low skeletal muscle mass) and a high mortality risk was shown in patients with sarcopenic obesity [16, 35].

Low serum creatinine (< 0.7 mg/dL) is a surrogate marker for frailty and sarcopenia (low skeletal muscle mass) particularly in the elderly; it is a strong predictor of mortality among patients with normal BMI in the setting of chronic diseases, for example coronary artery disease [36]. Interestingly, serum creatinine was found to modify the association of BMI (paradox) with mortality, as evident by the association of low creatinine levels with poor outcomes in hemodialysis patients [37]. In cancer patients, sarcopenic obesity was associated with the highest risk of mortality demonstrating that body mass composition can modify the association of BMI with survival; accordingly, the paradox was seen only when obesity was defined by BMI [35]. Serum creatinine levels were found to correlate with skeletal muscle mass measured by urinary creatine excretion/DEXA (Dual Energy X-ray Absorptiometry) in colorectal cancers patients as well as in healthy volunteers, albeit to a varying extent [38–42]. Nevertheless, serum creatinine to assess skeletal muscle mass indirectly represents a simple alternative when renal function is accounted for [41–43] and is especially relevant in advanced cancer where the growing metabolic demands of metastatic tumors lead to mobilization of nutrients from skeletal muscle [44]; we therefore included eGFR in the primary analysis to account for renal function. Less than 10% of the patients had prior history of CKD at baseline. Etiologies leading to low serum creatinine levels other than older age, female gender and the cancer-related catabolic state (e.g. rapid renal clearance, pregnancy, fever/acute illness and advanced liver disease) were unlikely to explain low serum creatinine levels in our cohort [45, 46].

### Gender-based difference in skeletal muscle mass, underlying sarcopenia and the impact on outcomes

A gender-based obesity paradox was recently demonstrated in the context of immunotherapy (anti-CTLA4/PD-1/PD-L1) and targeted therapy in metastatic melanoma [19]. In our study we also identified a predominantly male gender driven association of overweight/Class I obesity with lower risk of mortality/progressive disease; additionally, we found that this gender-based association was absent if serum creatinine was < 0.9 mg/dL. Gender-based density distribution of serum creatinine showed that most females had serum creatinine levels of < 0.9 mg/dL whereas the majority of males had serum creatinine > = 0.9 mg/dL, which was the threshold identified for serum creatinine in RSF for predicting outcomes and confirmed by Cox-PH analysis. Overweight/Class I obese patients had serum creatinine ≥0.9 mg/dL and were more commonly males. Class II/III obese patients had lower serum creatinine and were more commonly females in this study. Comparable gender-based differences in distribution of serum creatinine were previously shown in cancer patients [47]. Gender-based differences in body mass composition and/or muscle mass [48] are well documented and provide a potential biological basis for the association of low skeletal muscle mass with increased mortality risk in cancer patients [16, 49, 50] given that skeletal muscle is a large reservoir of proteins and other minerals/metabolites to meet the high requirements in a catabolic state such as in advanced cancer. Low muscle mass is associated with poor immune function as skeletal muscle provides key nutrients that are critical for lymphocyte and monocyte function [51, 52]) [53] which may be relevant in the setting of checkpoint based immunotherapy [54]. It is well documented that underlying sarcopenia and cachexia directly contribute to cancer-specific mortality [44].

In our study, serum creatinine levels above 0.7 mg/dL up to 0.9 mg/dL were also associated with worse outcomes although the highest risk was for patients with levels deemed to indicate sarcopenia clinically (< 0.7 g/dL) [36]. These findings suggest that extra muscle reserves could provide a survival advantage and an ability to cope in a stressful environment and the higher skeletal muscle mass in men may have offered a survival advantage in an advanced cancer setting. Preceding weight loss was unlikely to explain the findings as the relationship of BMI measured 3–6 months prior to treatment were similar to pre-treatment BMI based findings, indicating that the crossover of patients to lower weight categories had minimal impact on outcomes. Predictors of survival were in agreement with known clinical prognostic markers in melanoma [55]. To summarize, the hypothesized link between BMI, obesity, gender, serum creatinine, sarcopenia and outcomes in the context of PD-1 inhibition is illustrated in Additional file 1: Figure S8.

### Strengths and limitations

We used a data adaptive approach to derive cutoffs for defining BMI risk groups to analyze the data in an unbiased way. Given the underlying non-linear relationship of BMI and the complex interplay with other predictors of survival and progressive disease, adaptive tree-based methods (RSF/RF) were able to identify complex patterns and uncover higher order interactions with ease. Because the underlying philosophy of machine learning is prediction and not hypothesis testing, we confirmed the findings in the frequentist framework and such an integrated analytical approach is increasingly being adopted in clinical studies [56].

Information on diet and physical activity would have allowed a better exploration of potential mechanisms as it is known that serum creatinine levels can be affected by varying meat intake/diet [45], but is unlikely to affect the overall conclusions of the study given the setting of advanced metastatic cancer. Other unmeasured covariates could have contributed to the findings given the retrospective nature of the study. The sample size in the Class II/III obese group was limited in this study and therefore further studies in larger cohorts should assess whether these patients have a higher risk of progression and death in the setting of checkpoint inhibition as well as the impact of underlying sarcopenia/body mass composition on the observed relationship between BMI and survival. As serum creatinine is not the gold standard measure of skeletal muscle mass due to its relationship with renal and non-renal factors, the results are hypothesis generating and should be confirmed with DEXA or CT scan based skeletal muscle mass measurements in larger cohorts. While prior ipilimumab therapy in this cohort did not alter the main results as shown in the multi-variable analyses, the findings should be replicated in larger cohorts of patients treated with frontline PD-1 based checkpoint inhibition given the change in standard of care in melanoma. Our cohort included non-cutaneous melanomas and although we adjusted for this covariate in the multi-variable analyses, given the relatively low sample size of these rarer melanomas, the impact of BMI on survival may differ by subtypes and should be assessed in larger cohorts.

## Conclusions

In advanced melanoma patients treated with PD-1 based immune checkpoint inhibition (monotherapy/combination), overweight or Class I obesity was associated with a significantly lower risk of mortality and progressive disease compared to normal weight. These findings support the presence of the “obesity paradox” restricted to overweight/Class I obesity. The association was driven predominantly by overweight/Class I obese male patients. Lower serum creatinine levels (predominant among females), a surrogate for skeletal muscle mass in the setting of advanced cancer, was associated with worse survival outcomes and attenuated the gender-based obesity paradox finding. These observations suggest that sarcopenia (low skeletal muscle mass) or direct measures of body mass composition may be more suitable predictors of survival outcomes in the setting of PD-1 blockade in patients with melanoma and other cancers.

## Additional file

Additional file 1: Supplementary data showing additional exploratory and sensitivity analyses. (DOCX 2122 kb)
