# Supplementary material for: Complex inter-relationship of body mass index, gender and serum creatinine on survival: exploring the obesity paradox in melanoma patients treated with checkpoint inhibition
Source: J Immunother Cancer. 2019 Mar 29;7:89. doi: 10.1186/s40425-019-0512-5 (PMC6440018; doi:10.1186/s40425-019-0512-5)
Supplement: Supplementary file 1 — Supplementary data showing additional exploratory and sensitivity analyses. (DOCX 2122 kb) [file 40425_2019_512_MOESM1_ESM.docx]

**Additional file**

**Supplementary Figures**


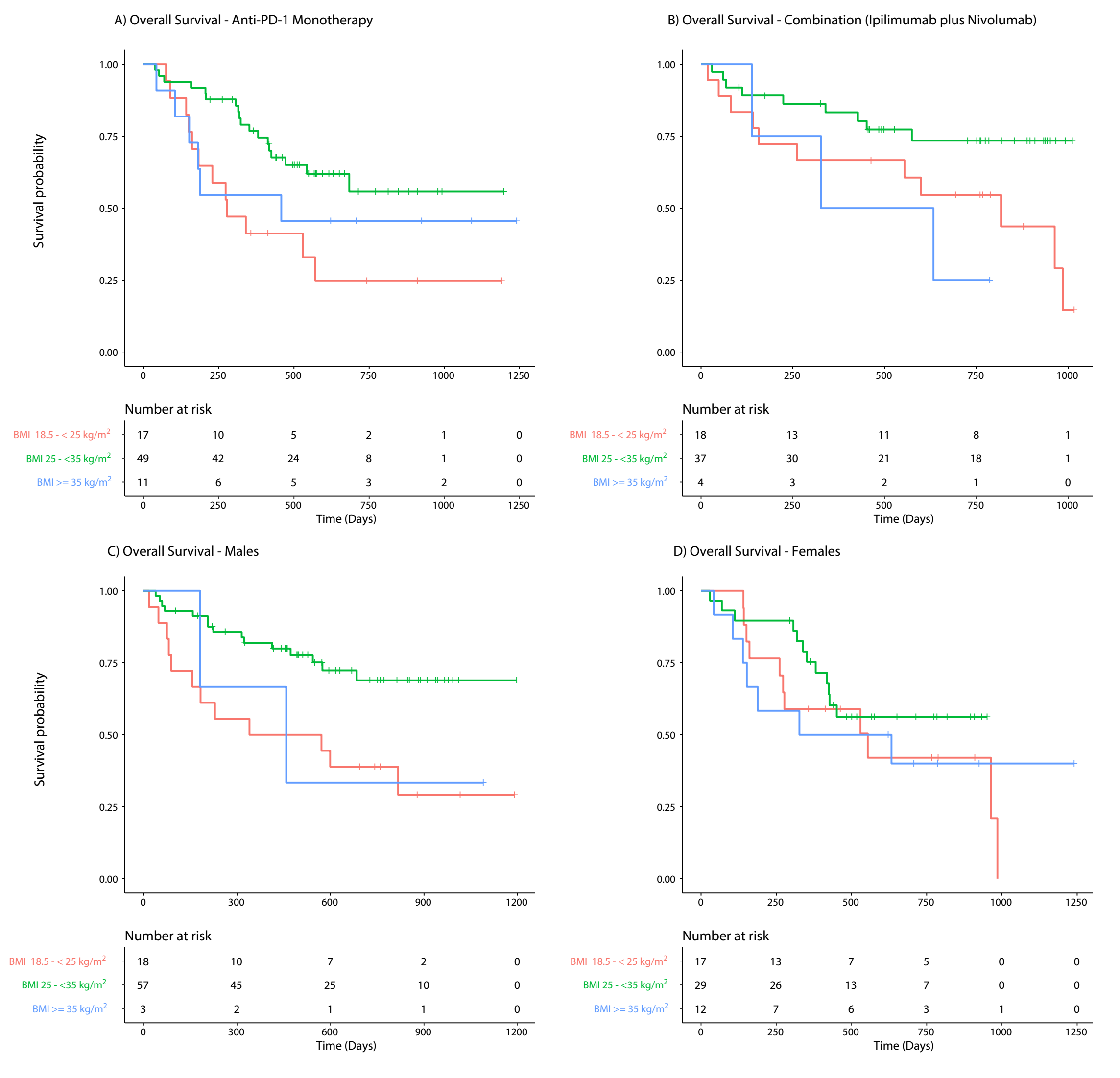


**Additional file 1: Figure S1. Kaplan-Meier survival curves for BMI and stratified by treatment and gender.**


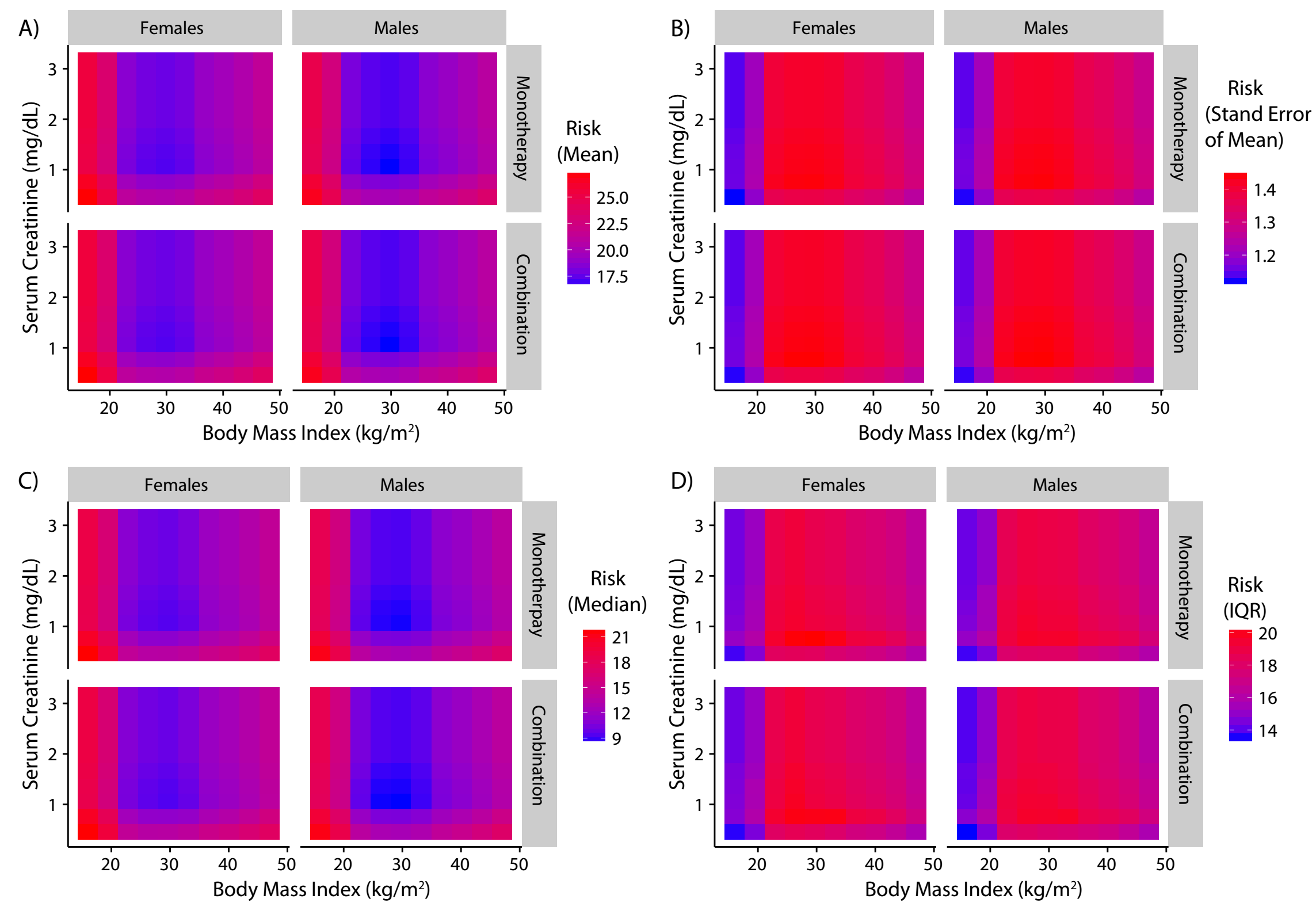


**Additional file 1: Figure S2. The mean (Panel A) and standard error of mean (Panel B), the median (Panel C) and IQR (Panel D) for the marginalized prediction function (Risk [OS]) is presented for examining the relationship and interaction between BMI, serum creatinine, gender and monotherapy or combination immunotherapy.**


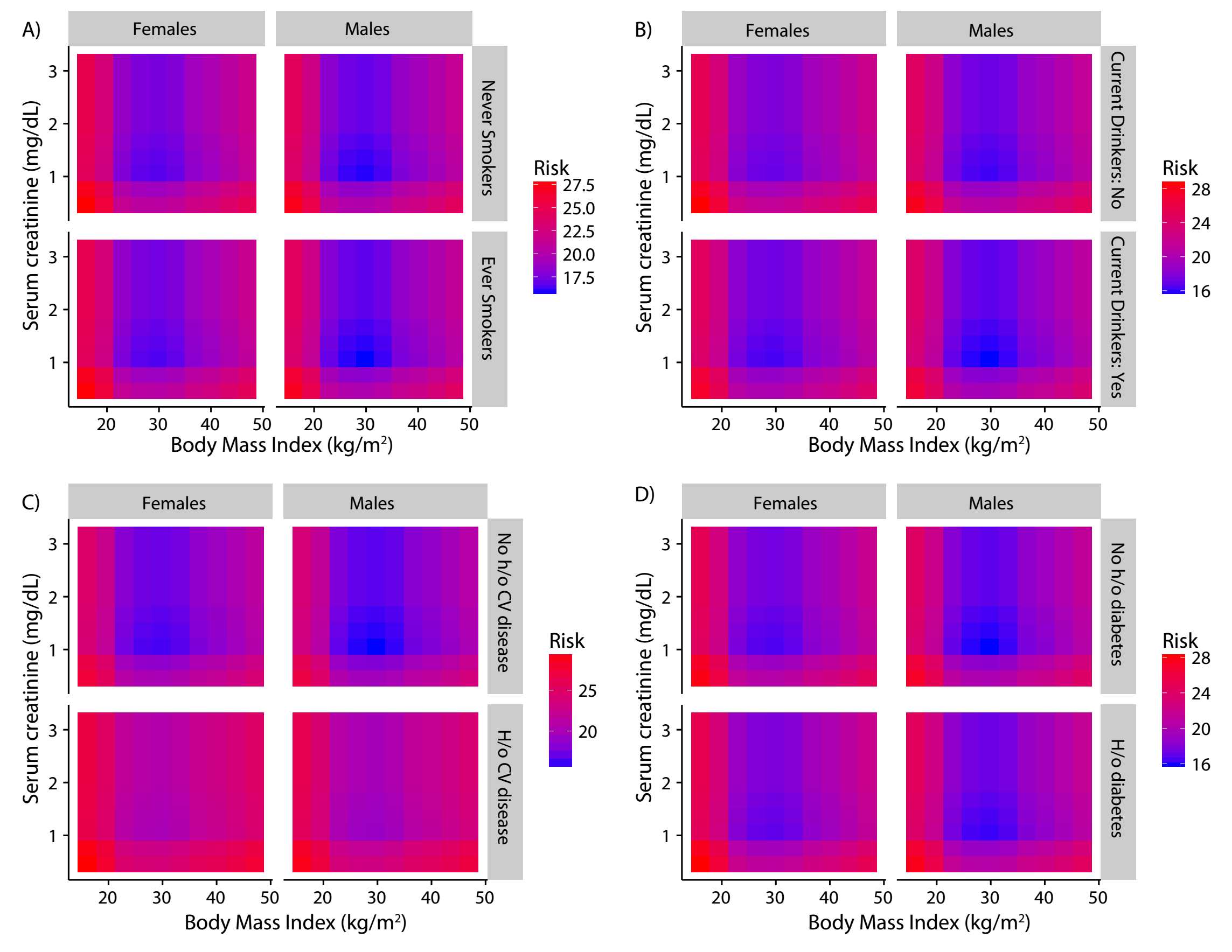


**Additional file 1: Figure S3. Exploration of gender-based interactions with BMI and serum creatinine: Lifestyle habits and co-morbidities (CV disease and diabetes).**


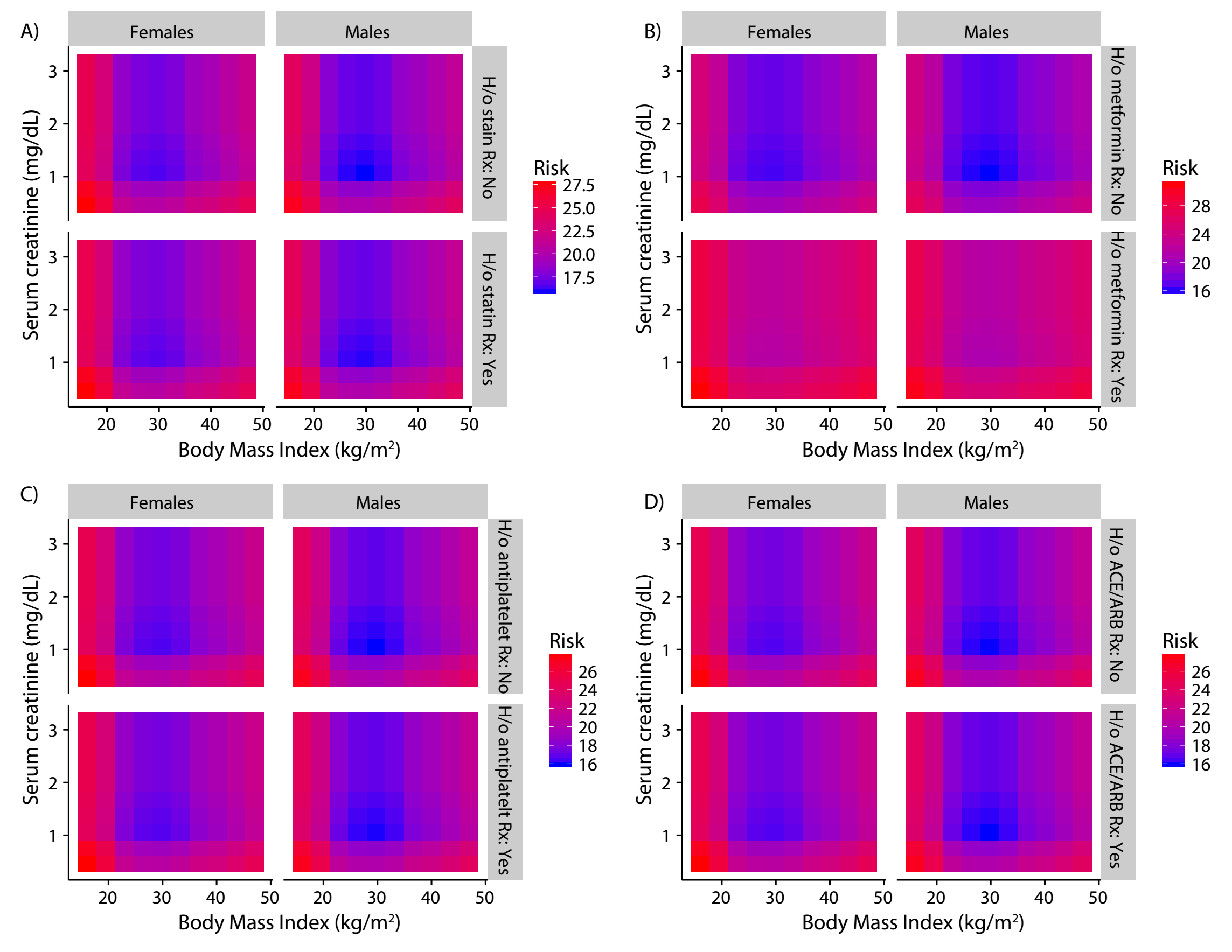


**Additional file 1: Figure S4. Exploration of gender-based interactions with BMI and serum creatinine: Co-medications for co-morbidities.**


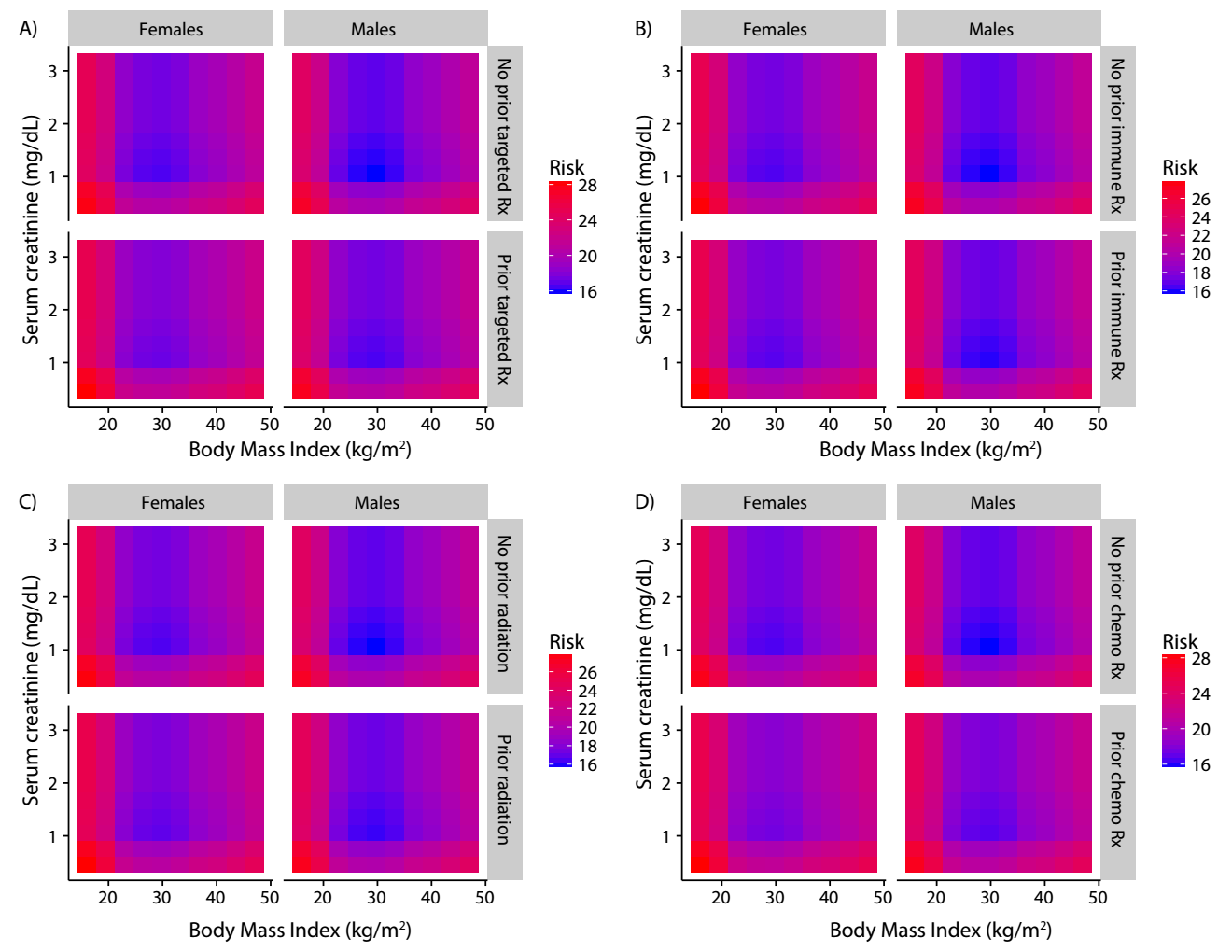


**Additional file 1: Figure S5. Exploration of gender-based interactions with BMI and serum creatinine: Prior treatments.**


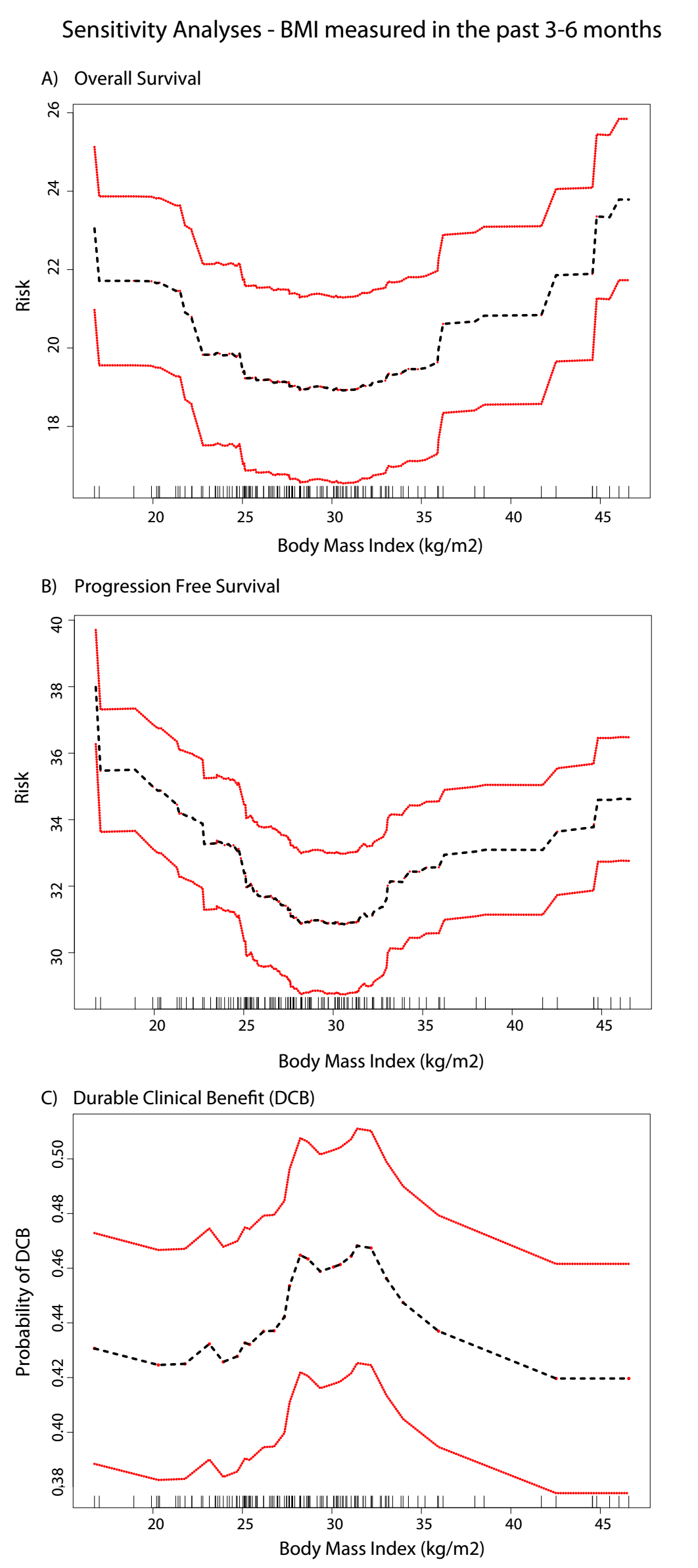


**Additional file 1: Figure S6. Sensitivity Analyses. BMI measured within the last 3-6 months and relationship with OS, PFS and DCB (RSF and RF analysis). Findings were similar to the results from primary analyses for pre-treatment BMI (refer Figure 1 for comparison).**


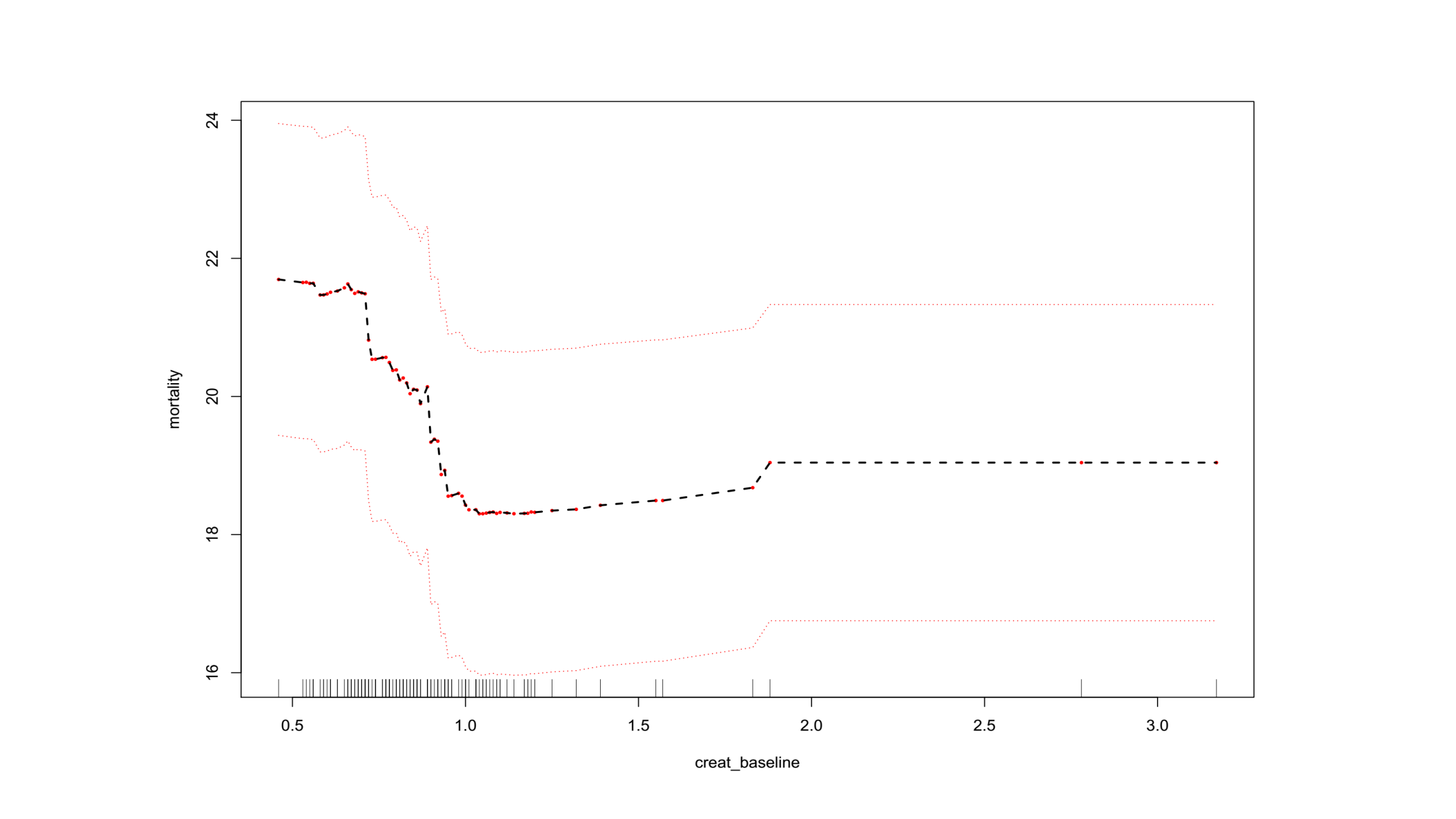


**Additional file 1: Figure S7. Relationship of serum creatinine on OS was similar when eGFR based on CKD-EPI was included (adjusted) as a continuous covariate.**


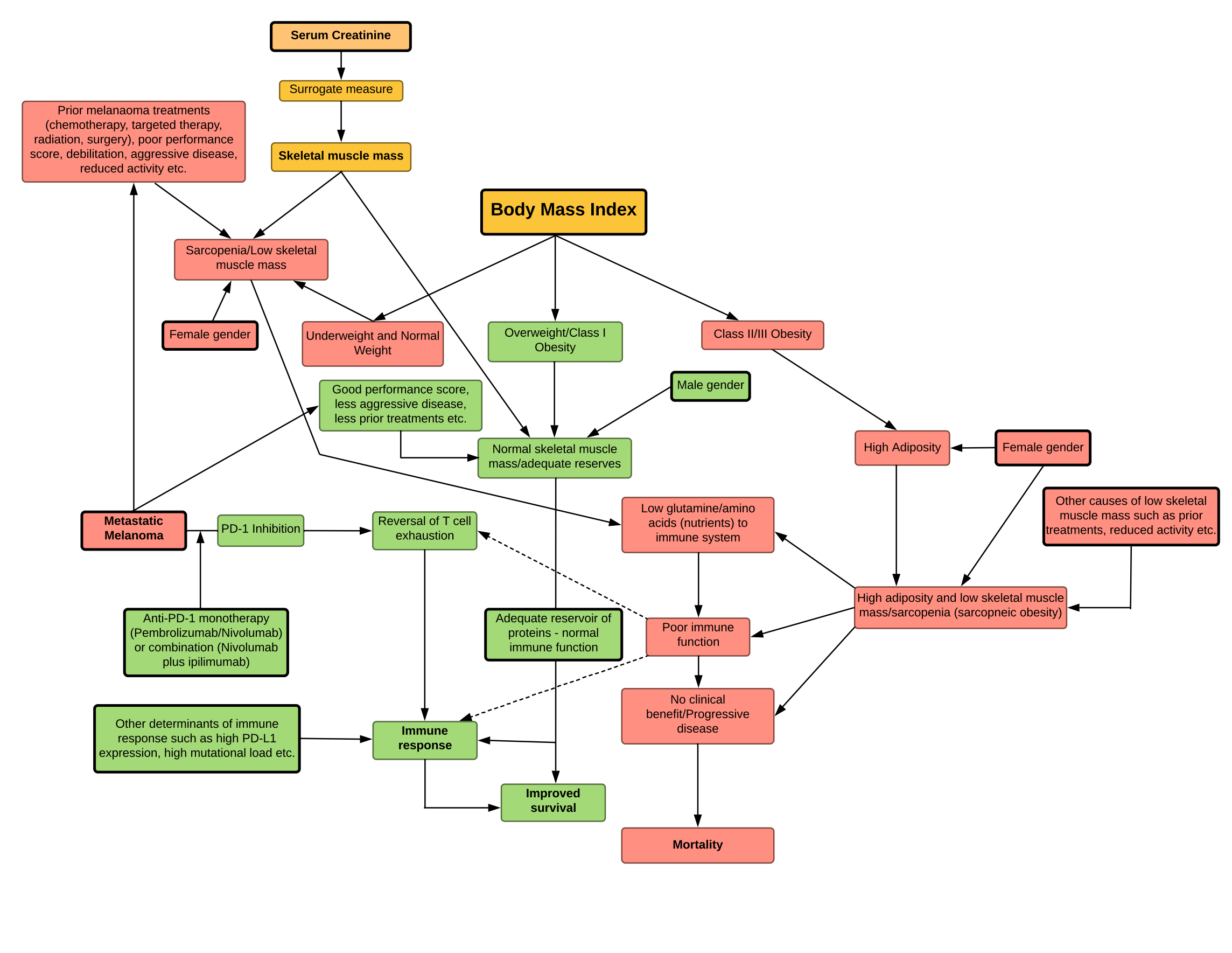


**Additional file 1: Figure S8. Illustration showing the hypothesized interplay of BMI, serum creatinine, sarcopenia and gender on survival outcomes in the context of PD-1 blockade.**

**Additional file 1: Table S1. Baseline characteristics by gender and serum creatinine for anti-PD-1 based immunotherapy treated patients.**

| **N = 139** | **Gender** | | **Serum Creatinine (mg/dL)** | |
| --- | --- | --- | --- | --- |
|  | **Females**  **N=60 (%)** | **Males**  **N=79 (%)** | **S. Cr.**  **< 0.9 mg/dL**  **N = 82 (%)** | **S. Cr.**  **≥0.9 mg/dL**  **N = 57 (%)** |
| **Demographics** |  |  |  |  |
| Age | 61.1 (13.2) | 64.4 (14.8) | 59.8 (13.1) | 64.5 (15.4) |
| Male gender | **-** | **-** | **29 (35.4)** | **50 (87.7)** |
| White race | 58 (96.7) | 78 (98.7) | 80 (97.6) | 56 (98.2) |
| Cutaneous Melanoma | 48 (80) | 73 (92.4) | 67 (81.7) | 54 (94.7) |
| BRAF mutation | 14 (23.3) | 21 (26.6) | 19 (23.2) | 16 (28.1) |
| NRAS mutation | 10 (16.7) | 4 (5.1) | 12 (14.6) | 2 (3.5) |
| **Disease severity** |  |  |  |  |
| Stage at baseline | IV M1c: 35 (58.3) | IV M1c: 45 (57) | IV M1c: 47 (57.3) | IV M1c: 33 (57.9) |
| Karnofsky Performance Score (KPS) | <70: 0 (0)  >=70: 60 (100) | <70: 4 (5.1)  >=70: 74 (9.5) N=78 | <70: 0 (0)  >=70: 81 (100)  N=81 | <70: 4 (7)  >=70: 53 (93) |
| LDH in U/L (Median and IQR; N=131) | 180.5 (145-287.5)  N=56/60 | 183 (146-261) N=75/79 | 187 (147 – 277)  N=78/82 | 177  (138 – 230)  N=53/57 |
| **Lifestyle habits** |  |  |  |  |
| Ever smokers | Former: 19 (31.7)  Current:6 (10) | Former: 38 (48.1)  Current: 5 (6.3) | Former: 27 (32.3)  Current: 8 (9.8) | Former: 30 (52.6)  Current: 3 (5.3) |
| Current drinkers | 30 (50) | 54 (68.4) | 44 (53.7) | 40 (70.2) |
| **Prior treatments** |  | |  |  |
| Immunotherapy | 26 (43.3) | 24 (30.4) | 35 (42.7) | 15 (26.3) |
| Chemotherapy | 6 (10) | 9 (11.4) | 10 (12.2) | 5 (8.8) |
| Radiation | 20 (33.3) | 18 (22.8) | 29 (35.4) | 9 (15.8) |
| Targeted therapy | 4 (6.7) | 6 (7.6) | 8 (9.8) | 2 (3.5) |
| **Co-morbidities** |  |  |  |  |
| Charlson’s Comorbidity Index (Mean and SD) | 7.9 (1.6) | 8.8 (2.1) | 8.1 (1.6) | 8.9 (2.2) |
| Diabetes | 7 (11.7) | 11 (13.9) | 11 (13.4) | 7 (12.3) |
| Hypertension | 23 (38.3) | 51 (64.6) | 39 (47.6) | 35 (61.4) |
| Hyperlipidemia | 16 (26.7) | 30 (38) | 25 (30.5) | 21 (36.8) |
| Chronic Kidney Disease (CKD) | 5 (8.3) | 6 (7.6) | 3 (3.7) | 8 (14) |
| Cardiovascular disease (CAD/CHF/MI/AF) ^#^ | 6 (10) | 15 (19) | 8 (9.8) | 13 (22.8) |
| Autoimmune/Immune mediated disorders | 8 (13.3) | 9 (11.4) | 10 (12.2) | 7 (12.3) |
| **Co-medications for co-morbidities** |  | |  |  |
| Anti-platelet agents (Aspirin/Clopidogrel) | 11 (18.3) | 25 (31.6) | 16 (19.5) | 20 (35.1) |
| Anti-hypertensive medications (any) | 24 (40) | 44 (56) | 33 (40.2) | 35 (61.4) |
| ACE or ARB inhibitors | 11 (18.3) | 28 (35.4) | 15 (18.3) | 24 (42.1) |
| Metformin | 4 (6.7) | 8 (10.1) | 8 (9.8) | 4 (7) |
| Statins | 11 (18.3) | 24 (30.4) | 18 (22) | 17 (29.8) |
| Oral Steroids | 3 (5) | 9 (11.4) | 8 (9.8) | 4 (7) |
| **Clinical chemistry and Vitals** |  | |  |  |
| Albumin in g/dL (Mean and SD) | 4 (0.5) | 4 (0.47) | 4 (0.47) | 4 (0.49) |
| ANC K/uL (Mean and SD) | 4.7 (2.1) | 5.3 (2.4) | 4.9 (2.3) | 5.3 (2.3) |
| ALC K/uL (Mean and SD) | 1.6 (0.8) | 1.5 (1.4) | 1.6 (1.4) | 1.4 (0.7) |
| Hemoglobin g/dL (Mean and SD) | 12.4 (1.4) | 13.1 (1.9) | 12.6 (1.6) | 13.1 (1.9) |
| Serum Creatinine mg/dL (Mean and SD) | **0.78 (0.3)** | **1 (0.3)** | **0.73 (0.1)** | **1.16 (0.4)** |
| eGFR^ (ml/min/1.73m^2^) | >=60: 56  <60: 4 | >=60: 70  <60: 9 | >=60: 82; <60: 0 | >=60:44; <60: 13 |
| eGFR by CKD-EPI equation in ml/min/1.73m^2^ (Median and IQR) | 90.38 (77.9 – 100.3) | 83.62 (69.62 – 92.65) | 93.16 (86.91-101.3) | 72.12 (58.14 – 83.26) |
| Fasting Glucose in mg/dL (Median and IQR) | 101.5 (93.5-113) | 105 (94-126) | 102 (92-115) | 106 (95-122) |
| BMI at baseline (kg/m^2^) | 29.3 (7.1) | 28 (4.3) | 28.5 (6.4) | 28.6 (4.6) |
| Alkaline Phosphatase in U/L (Median and IQR) | 70.5 (59 - 97) | 75 (63-95) | 75.5 (61-95) | 71 (62-98) |
| ALT in U/L (Median and IQR) | 15 (11-22.5) | 18 (13-25) | 17 (11-24) | 16 (12-23) |
| AST in U/L (Median and IQR) | 16 (12.5-21) | 20 (14-28) | 18 (13-25) | 19 (13-25) |
| Systolic blood pressure in mm Hg (Mean/SD) | 129 (20.3) | 133.4 (17.3) | 131.4 (19.3) | 131.7 (17.9) |
| Diastolic blood pressure in mm Hg (Mean/SD) | 74.3 (12.7) | 78.8 (12.3) | 77 (12) | 76.7 (13.6) |
| **Disease related weight loss** |  |  |  |  |
| BMI measured up to 6 months before baseline (Mean and SD) | 29.5 (7.4)  N=58/60 | 28. 3 (4.1)  N=73/79 | 28.8 (6.6)  (N=79/82) | 29 (4.3)  (N=52/57) |
| **Treatment** |  |  |  |  |
| Anti-PD-1 immunotherapy type | Mono: 34 (56.7)  Combination: 26 (43.3) | Mono: 45 (57)  Combination: 34 (43) | Mono: 47 (57.3)  Combination: 35 (42.7) | Mono: 32 (56.1)  Combination: 25 (43.4) |

***Includes three patients with BMI < 18.5 for descriptive purposes. Analyses by Cox-PH/logistic regression was performed by excluding underweight patients (n=3) but were included for RSF analysis where BMI was included as a continuous variable.**

**^eGFR – Estimated Glomerular Filtration Rate (Cockcroft-Gault).**

**# CAD: Coronary Artery Disease; CHF: Congestive Heart Failure; MI: Myocardial Infarction and AF: Atrial Fibrillation**

**IQR: Inter Quartile Range**

**Additional file Text**

**Methods**

Random Survival Forests – an ensemble machine learning algorithm which is an extension of random forests suitable for handling right censored outcome (1,2).

VIMP – is the prediction error of the original constructed ensemble minus the ensemble constructed newly obtained by randomizing “x” assignment (1,2).

Minimal depth – is the distance of the splitting variable from the root of the trunk (1,2).

Concordance index – fraction of all pairs of subjects whose predicted survival times can be correctly ordered that can be ordered among all subjects (1).

Marginalized predication functions and their measures of dispersion were plotted (as heat maps) based on the algorithm detailed in Goldstein et al., 2015 (4) and implemented in “mlr” package (5) and the “mmpf” package (6). Cox-Proportional Hazards modeling was performed using STATA version 13.

**Results**

*Exploration of other complex interactions for explaining gender-based association of overweight/Class I obesity with improved outcomes*

Cardiovascular comorbidities abolished the obesity paradox pattern, presence of diabetes or hyperlipidemia largely attenuated the paradox but did not provide a strong basis for explaining gender-based difference in outcomes (Supplementary Fig-3). For lifestyle habits, there was no gender-based interaction with smoking (Supplementary Fig-3). There were no obvious interactions with different anti-hypertensive drugs, statins or anti-platelet agents but patients receiving metformin had worse survival (Supplementary Fig-4) although there were few patients who received metformin and did not provide an explanation for the gender-based association.

Among prior treatments, patients with prior history of targeted therapy and chemotherapy had worse outcomes in general but relatively few patients had these prior treatments and did not explain the gender-based difference in outcomes (Supplementary Fig-5 and Table-1). There was no gender-based interaction noted however for BRAF and NRAS mutation for the association of BMI with outcomes (Figure not shown; Refer Supplementary Table-1 for baseline differences). Exploration of other interactions of BMI with covariates related to demographics, disease severity showed that overweight/Class I obese patients were more likely to have improved survival outcomes if they were aged 45 -75 years, had Charlson’s Comorbidity Index of <10, KPS >=80, normal LDH levels and Stage < IVM1c (Figure not shown; Refer Supplementary Table-1 for differences in baseline characteristics for these variables) but these findings do not provide an explanation for a gender-based difference in outcomes. The obesity paradox was attenuated in the presence of low albumin, high ANC, abnormal ALC, low hemoglobin (Figure not shown; Refer Supplementary Table-1 for baseline differences). These results concur with findings from studies on clinical prognostic markers in melanoma (3). Patients with lower serum creatinine were more likely to have lower hemoglobin and serum albumin (Supplementary Table-1) and although these factors likely have contributed to the findings, gender based density distributions of albumin and hemoglobin within BMI risk groups and creatinine groups show that albumin and hemoglobin on their own do not explain the gender based differences in survival outcomes as strongly as serum creatinine and the absolute numbers of patients who had hemoglobin and serum albumin below the risk threshold were relatively low (Supplementary Table-1).

**Additional file References**

1. Ishwaran H, Kogalur UB, Blackstone EH, Lauer MS. Random survival forests. Ann Appl Stat. 2008 Sep;2(3):841–60.

2. Breiman L. Random Forests. Mach Learn. 2001 Oct 1;45(1):5–32.

3. Eton O, Legha SS, Moon TE, Buzaid AC, Papadopoulos NE, Plager C, et al. Prognostic factors for survival of patients treated systemically for disseminated melanoma. J Clin Oncol Off J Am Soc Clin Oncol. 1998 Mar;16(3):1103–11.

4. Goldstein, A. Kapelner, J. Bleich, and E. Pitkin. Peeking inside the black box: Visualizing statistical

learning with plots of individual conditional expectation. Journal of Computational and Graphical

Statistics, 24(1):44–65, 2015.

1. B. Bischl, M. Lang, L. Kotthoff, J. Schiffner, J. Richter, E. Studerus, G. Casalicchio, and Z. M. Jones. mlr: Machine learning in R. Journal of Machine Learning Research, 17(170):1–5, 2016.
2. Z.M. Jones. mmpf: Monte-Carlo Methods for Prediction Functions. The R Journal Vol 10/1, July 2018.
